# Supplementary material for: Genetic tuning of retinal ganglion cell subtype identity to drive visual behavior
Source: Nat Commun. 2025 Sep 30;16:8678. doi: 10.1038/s41467-025-63675-w (PMC12484735; doi:10.1038/s41467-025-63675-w)
Supplement: Supplementary file 1 — Supplementary Information [file 41467_2025_63675_MOESM1_ESM.pdf]

Genetic tuning of retinal ganglion cell subtype identity to drive visual behavior

Marcos L. Aranda[1], Jacob D. Bhoi[1], Omar A. Payán Parra[1], Seul Ki Lee[1], Tomoko Yamada[1],

Yue Yang[1], Tiffany M. Schmidt[1,2*]

[1] Department of Neurobiology, Northwestern University, Evanston, IL, USA.

[2] Department of Ophthalmology, Feinberg, School of Medicine, Northwestern University, Chicago, IL, USA.

*Corresponding author: Email: tiffany.schmidt@northwestern.edu

Supplementary information

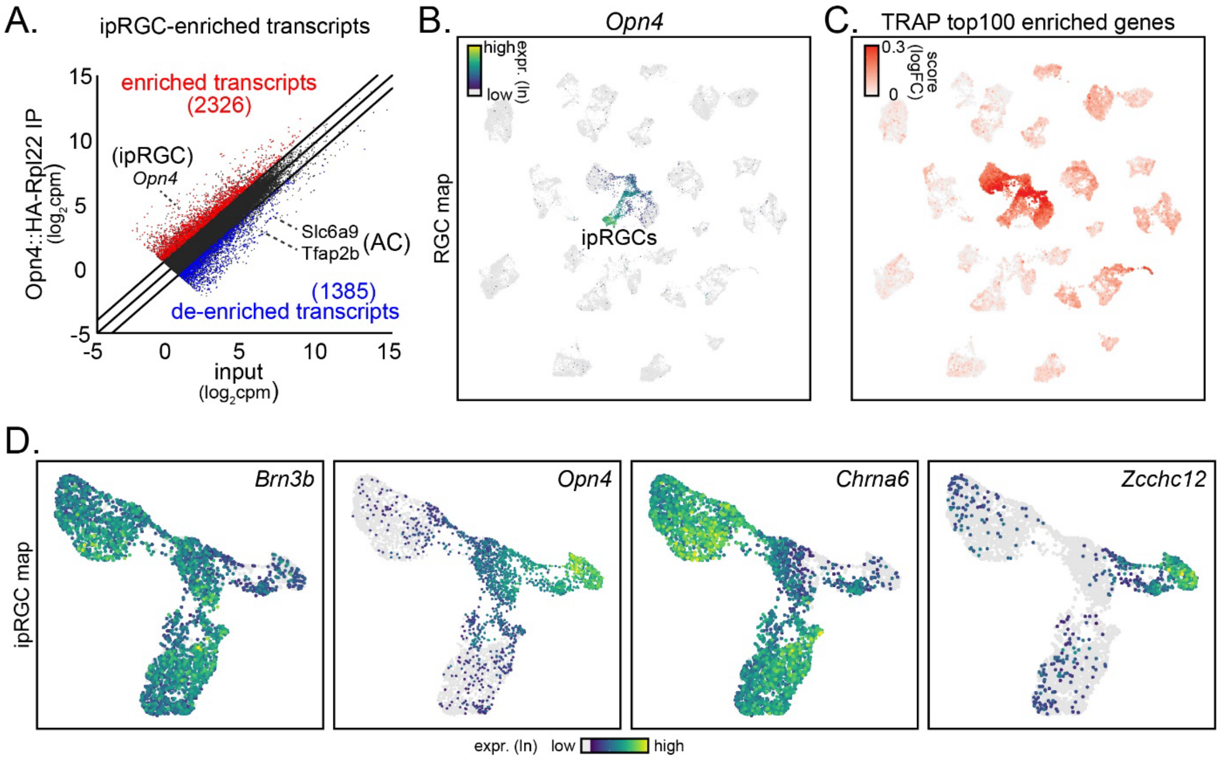

Supplementary Figure 1. Profiling the ipRGC transcriptome using TRAP. (A) Immunoprecipitation of HA-Rpl22 expressed selectively in *Opn4*$^{Cre/+}$ retinal cells followed by sequencing of ribosome-bound mRNA and total retinal mRNA reveals enrichment and de-enrichment of thousands of transcripts as indicated in red or blue, respectively (n = 2-4 biological replicates, log2FC>1, FDR<0.05). Among enriched transcripts include the ipRGC gene marker Opn4 and among de-enriched transcripts include the amacrine cell (AC) gene markers *Slc6a9* and *Tfap2b*. (B, C) *Opn4* mRNA expression (B) and the top 100 genes isolated from *Opn4*$^{Cre/+}$ *;* *Rpl22*^{HA} retinal cells that are enriched in ipRGCs, among all RGC cell types (C). (D) *Brn3b*, *Opn4*, *Chrna6* and *Zcchc12* mRNA expression in ipRGC clusters.

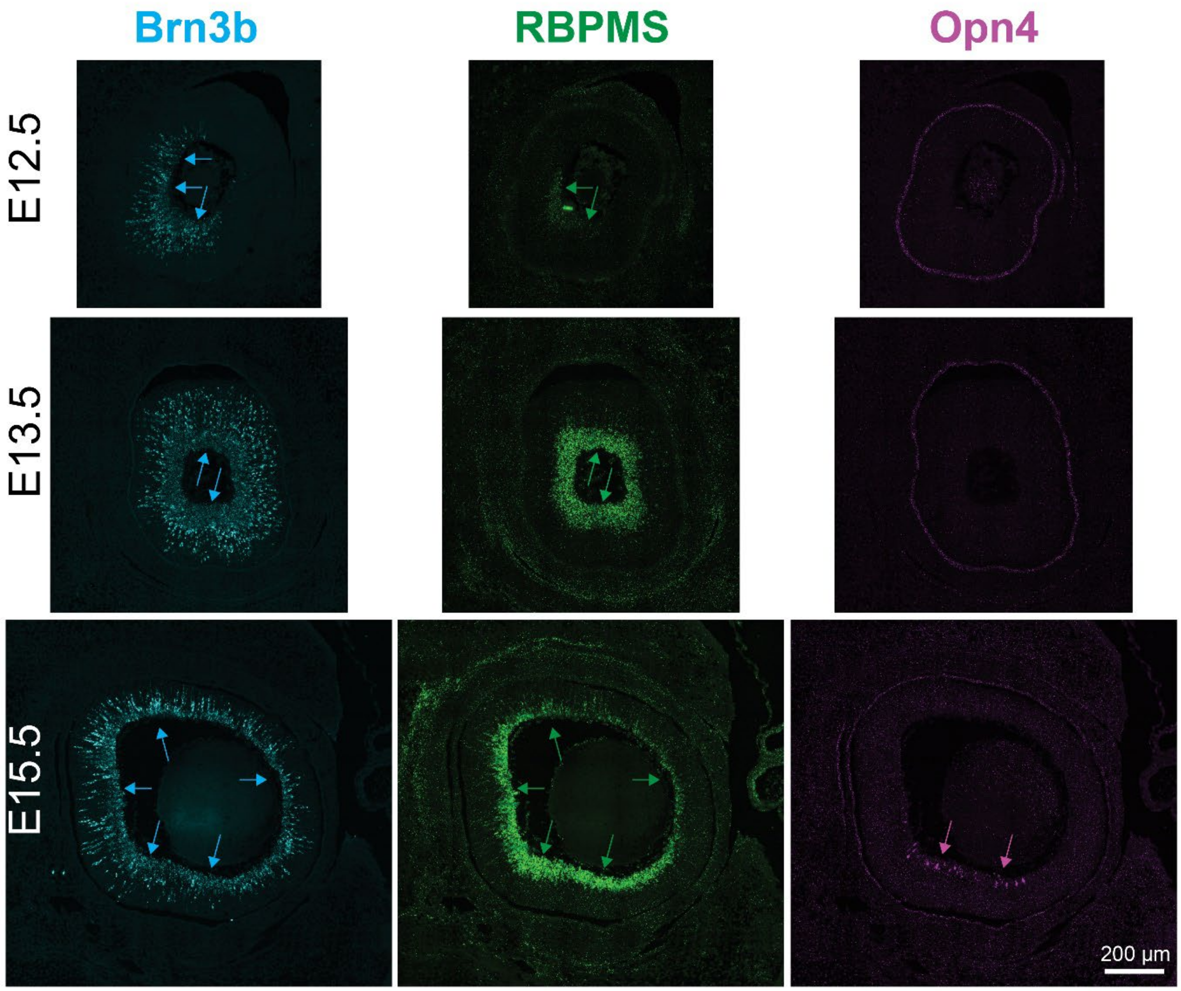

Supplementary Figure 2. *Opn4* is expressed after RGC specification. RNAscope fluorescent in situ hybridization (FISH) labeling in developing eyecups for *Brn3b* (cyan arrows), *Rbpms* (green arrows), and *Opn4* (magenta arrows) mRNA at various embryonic stages. *Opn4* expression is undetectable until E15.5, after RGC specification.

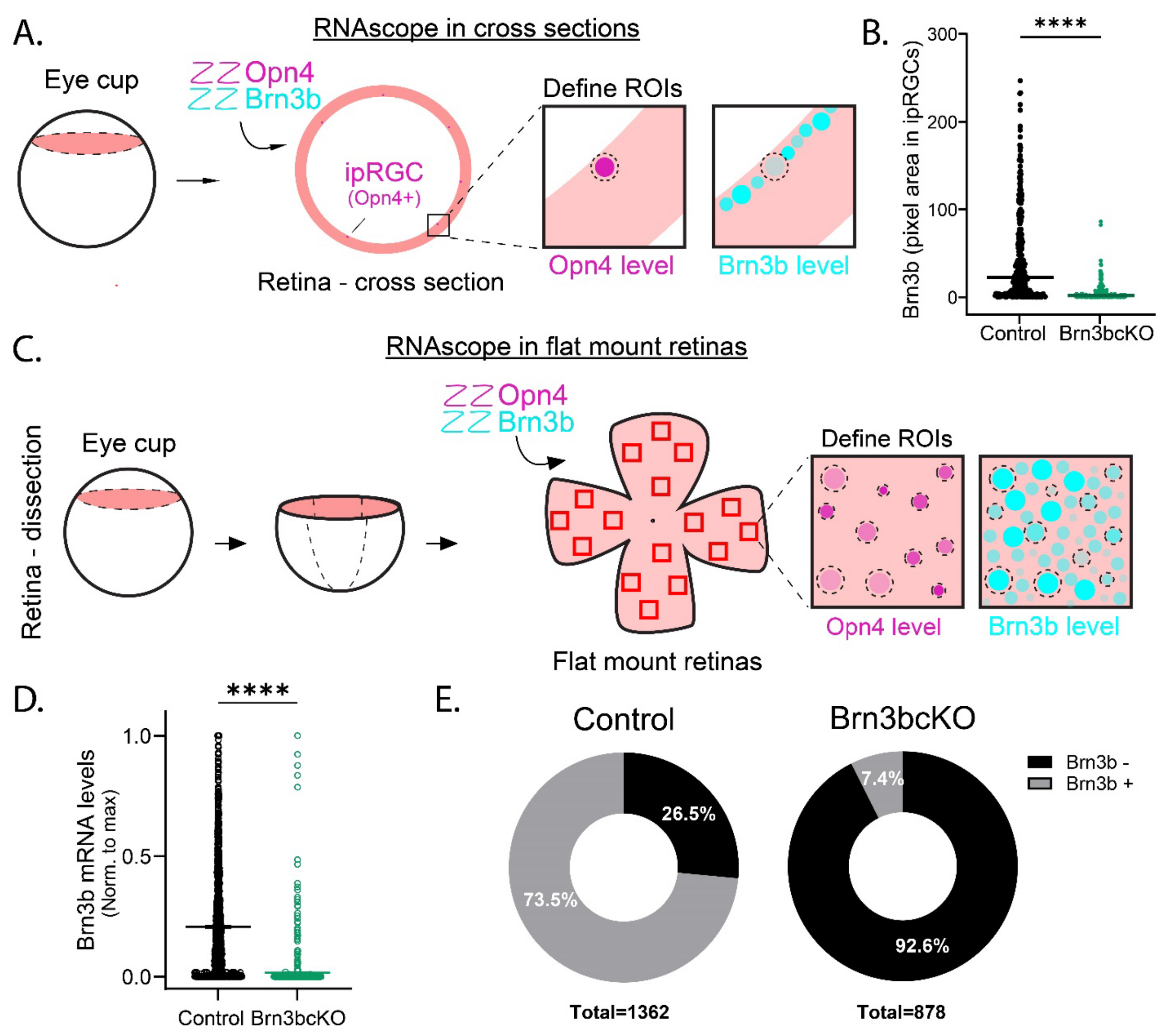

Supplementary Figure 3. Brn3bcKO mice present decreased Brn3b mRNA expression in ipRGCs. (A) Schematic representation of RNAscope in cross-sectioned retinas. (B) *Brn3b* mRNA levels are significantly decreased in ipRGCs of Brn3bcKO (green, n=216) mice compared to control (black, n=336 cells) littermates (P=0.0001). (C) Schematic representation of RNAscope in flat mount retinas. ROIs: region of interest. (D) *Opn4* mRNA levels are significantly increased in ipRGCs Brn3bcKO (green, n=878) compared to control (black, n=1362 cells) littermates (P=0.0001). (E) Proportion of *Brn3b*-negative (Brn3b-) and *Brn3b*-positive (Brn3b+) mRNA in

ipRGCs from control and Brn3bcKO mice measured by RNAscope. Source data are provided as a Source Data file. Lines are median values, ****$P<0.001$, two-tailed Mann Whitney U test.

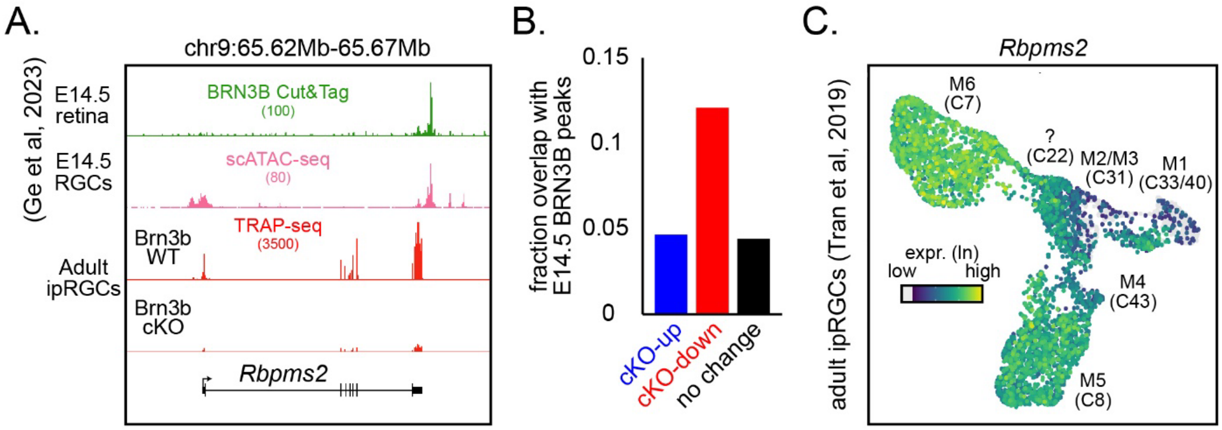

Supplementary Figure 4. BRN3B binding is enriched at Brn3bcKO downregulated genes. (**A**) UCSC genome browser tracks of BRN3B Cut&Tag, scATAC-seq [21], and TRAP-seq data at the *Rbpms2* locus. *Rbpms2*, which is robustly downregulated in ipRGCs upon Brn3bcKO, harbors an ATAC-seq peak downstream of its gene body that is bound by BRN3B. (**B**) Genes that are downregulated upon Brn3bcKO in ipRGCs show enrichment for BRN3B peaks compared to genes that are upregulated or not changed upon Brn3bcKO. (**C**) *Rbpms2* mRNA expression in ipRGC clusters. Source data are provided as a Source Data file.

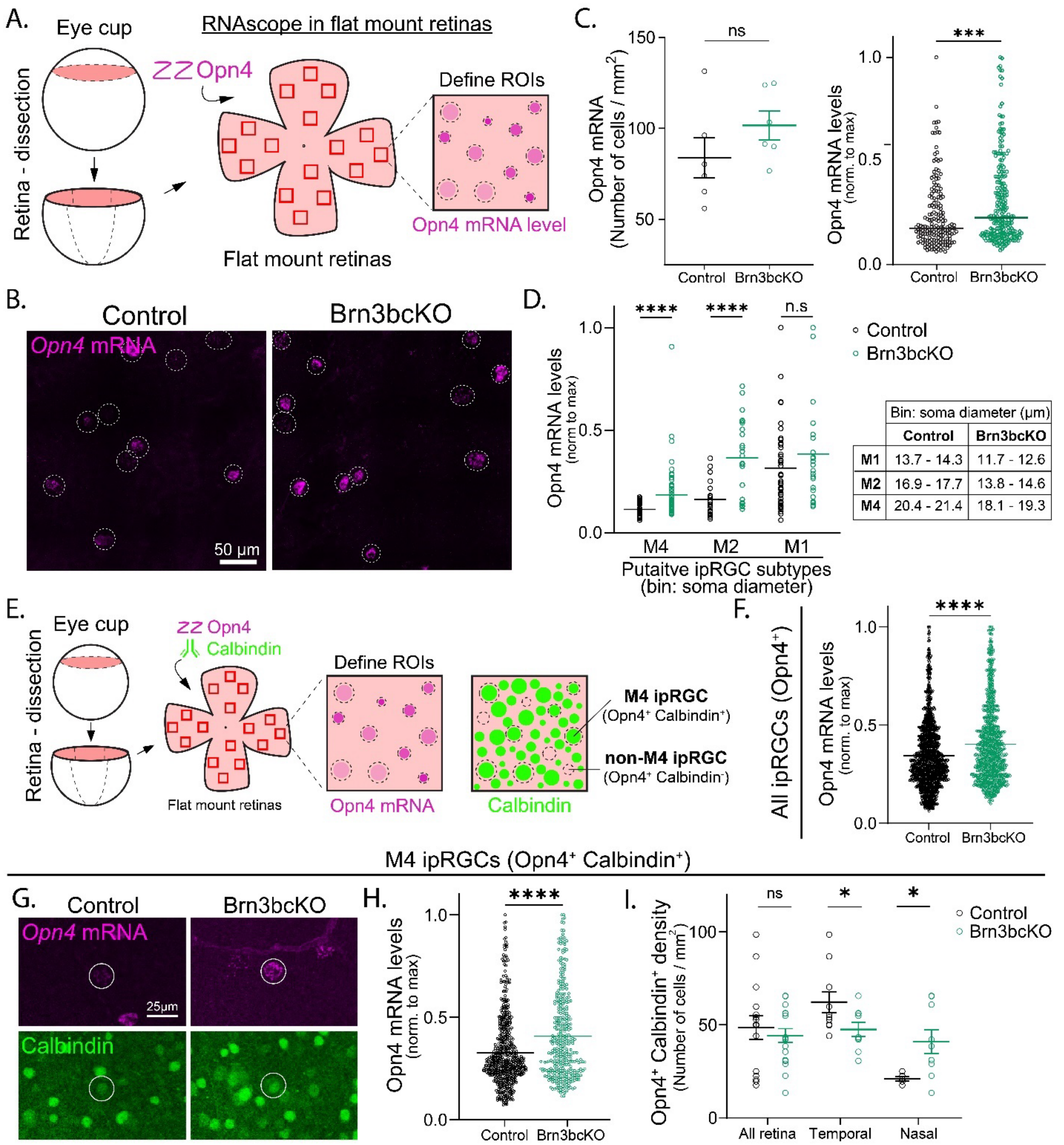

Supplementary Figure 5. Brn3bcKO mice present increased *Opn4* mRNA expression in ipRGCs. (A) Schematic representation RNAscope in flat-mount retinas using *Opn4* mRNA probe. ROIs: region of interest. (B) Representative images of *Opn4* mRNA labeling in flat mount retinas. (C) Left: density of *Opn4* mRNA-expressing cells (n=6 mice per group) (P=0.219). Right: *Opn4* mRNA levels are significantly increased in ipRGCs of Brn3bcKO mice (green, n=182 cells)

compared to control littermates (black, n =242 cells) (P=0.0002). (D) Left: *Opn4* mRNA levels are significantly increased in M4 (n= 33 cells in Control; N=60 cells in Brn3bcKO, P=0.0001), M2 (n= 22 cells in Control; N=24 cells in Brn3bcKO, P=0.0001) ipRGCs of Brn3bcKO mice (green) compared to control (black) littermates. No differences were observed in putative M1 ipRGCs (n= 44 cells in Control; N=22 cells in Brn3bcKO, P=0.201). Right: soma diameter bins to classify ipRGC subtypes. (E) Schematic representation of RNAscope in flat-mount retinas using *Opn4* mRNA probe and anti-calbindin antibody. (F) *Opn4* mRNA levels are significantly increased in all ipRGCs of Brn3bcKO mice (green, n=889 cells) compared to control littermates (black, n=1362 cells) (P=0.00001). (G) Representative images of *Opn4* mRNA and calbindin expressing cells in control and Brn3bcKO mice. (H) *Opn4* mRNA levels are significantly increased in M4 ipRGCs (*Opn4*$^{+}$ calbindin^{+}) of Brn3bcKO mice (green, n=379 cells) compared to control littermates (black, n=593 cells) (P=0.00001). (I) density of *Opn4*$^{+}$ calbindin^{+}-expressing cells (M4 ipRGCs; All retina: N=19 in Control, N=14 in Brn3bcKO, P=0.551; Temporal: N=10 in Control, N=9 in Brn3bcKO, P=0.048; Nasal: N=5 in Control, N=9 in Brn3bcKO, P=0.043). Source data are provided as a Source Data file. Lines are median values, n.s. (not significant) P>0.05, *P<0.05, ***P<0.001, ****P<0.0001, two-tailed Student's t- and Mann Whitney U tests.

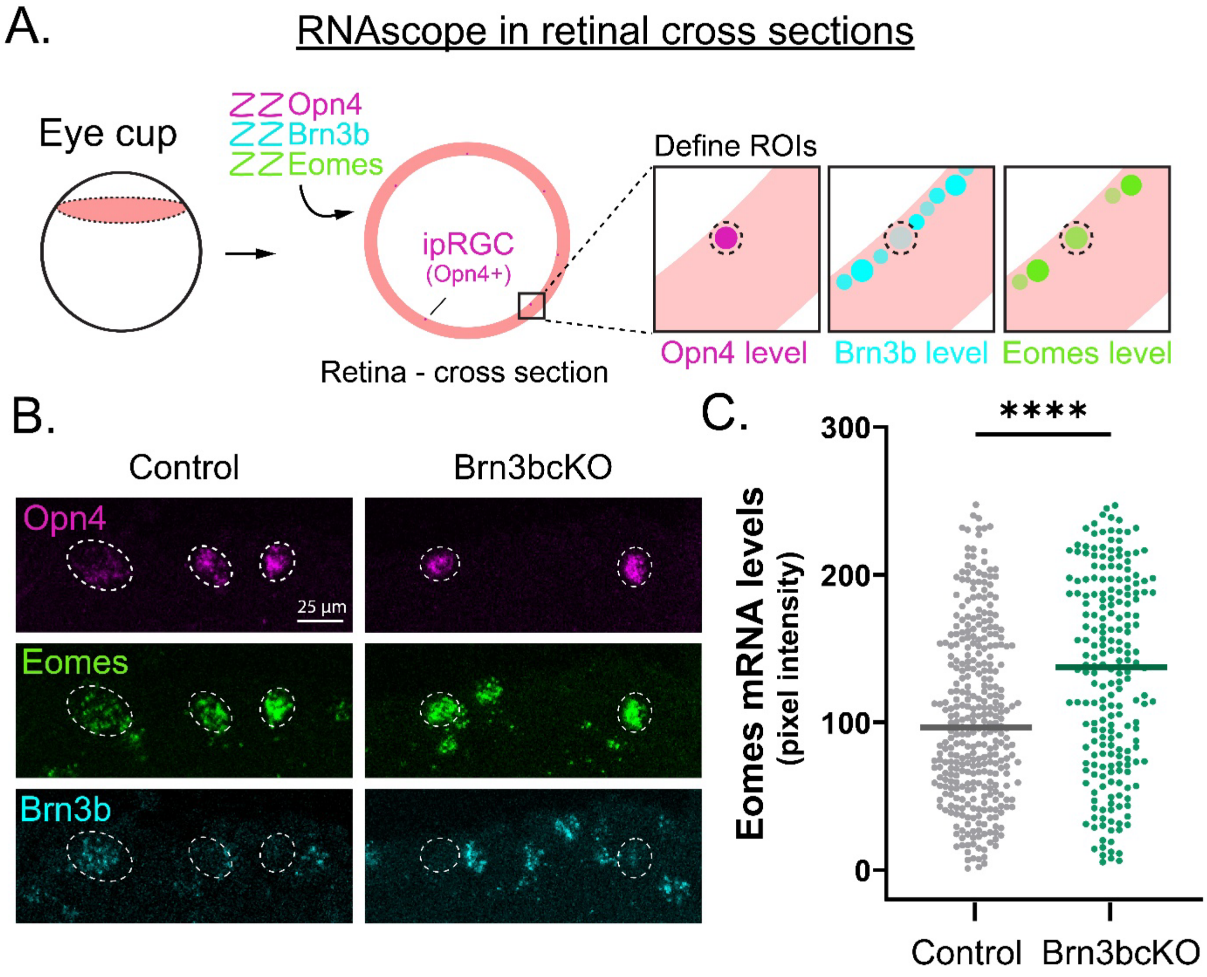

Supplementary Figure 6. Brn3bcKO mice present increased *Eomes* mRNA expression in ipRGCs. (A) Schematic representation of RNAscope in cross-sectioned retinas. ROIs: region of interest. (B) *Opn4, Eomes* and *Brn3b* mRNA expression in ipRGCs (dashed ellipse) form control (*Opn4*$^{Cre/+}$; *Brn3b*$^{+/+}$) and Brn3bcKO retinal sections. (C) *Eomes* mRNA levels are significantly increased in ipRGCs Brn3bcKO (green, n=216 cells) compared to control (grey, n=336 cells) littermates (P=0.00001). Source data are provided as a Source Data file. Lines are median values, ****P<0.0001, two-tailed Mann Whitney U test.

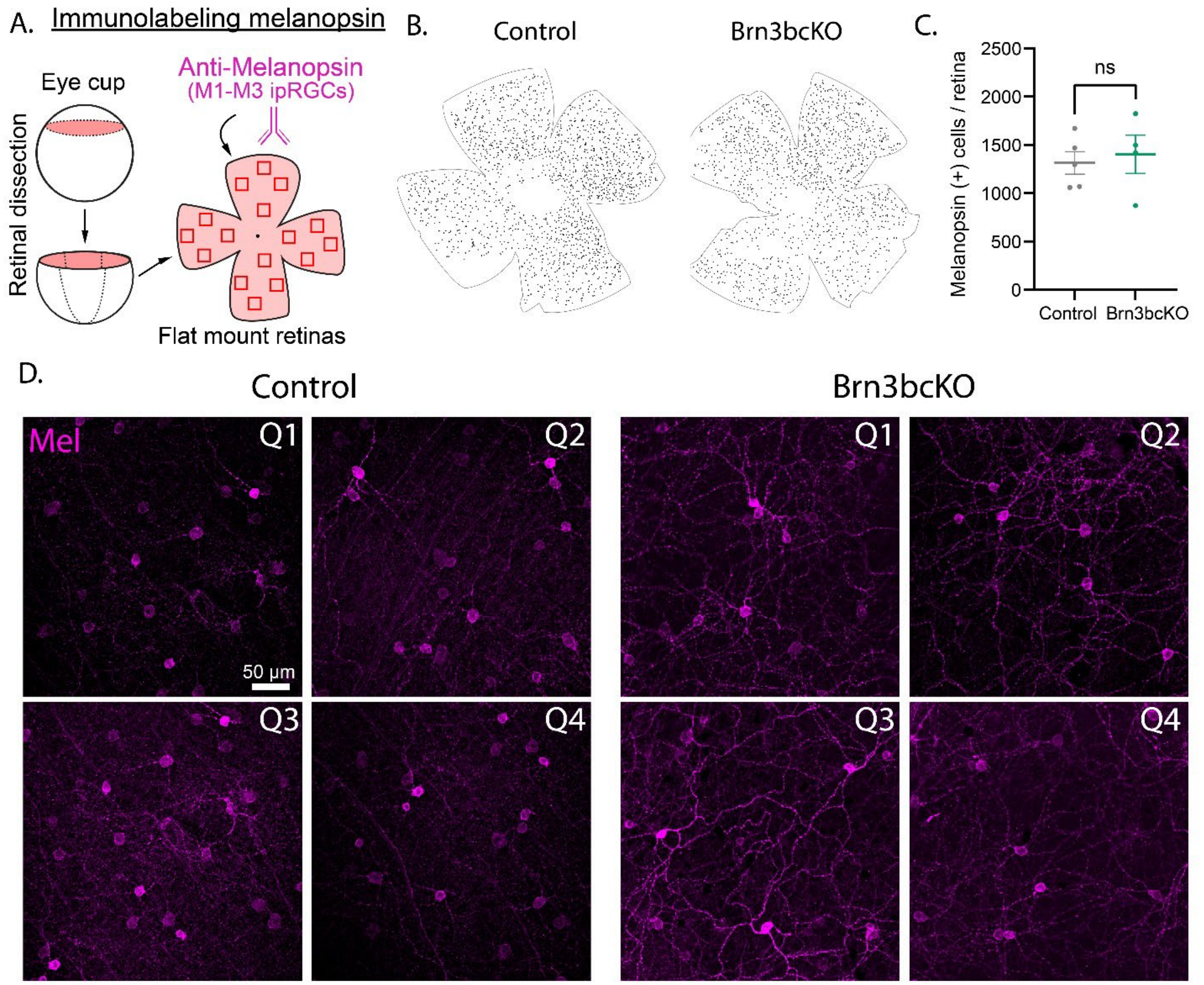

Supplementary Figure 7. Melanopsin levels in flat mount retinas. (A) Schematic representation melanopsin immunolabeling in flat-mount retinas. (B) Representative quantification of total number of melanopsin-positive cells (each dot represents a cell) in control and Brn3bcKO retinas. (C) No differences in the total number of melanopsin-positive cells were observed between control (grey, n=5 retinas) and Brn3bcKO (green, n=4 retinas) mice (P=0.696). (D) Representative images of melanopsin immunolabeling from different quadrants (Q1-Q2) of adult control and Brn3bcKO retinas. Source data are provided as a Source Data file. Lines are median (B-C), n.s. (not significant) P>0.05, two-tailed Mann Whitney U test.

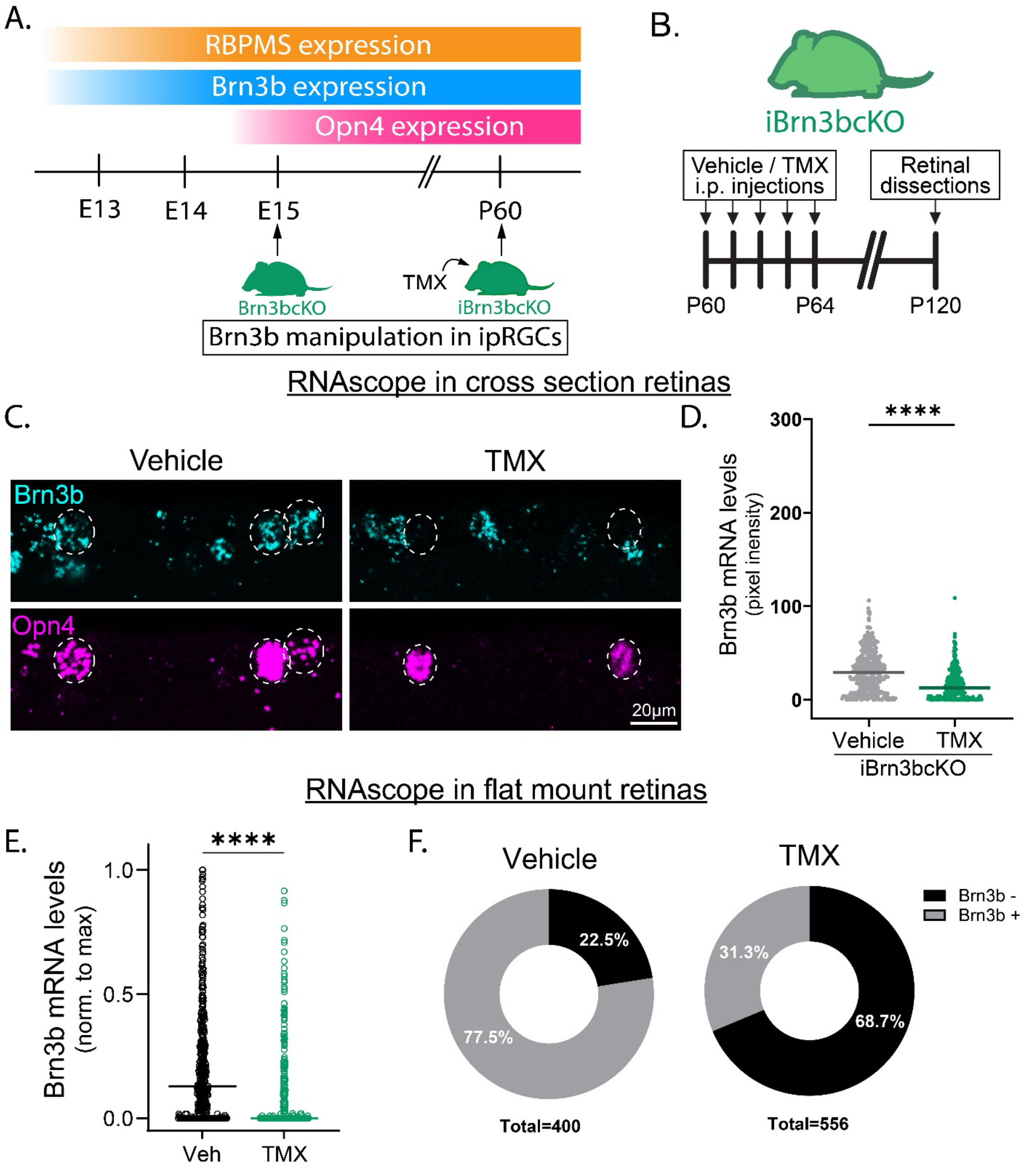

Supplementary Figure 8. Characterization of inducible-Brn3bcKO (iBrn3bcKO) mice. (A) Schematic representation of RBPM *Brn3b* and *Opn4* expression during development. (B) Schematic protocol to induce Cre-recombinase expression in iBrn3bcKO mice. (C) Representative

images of RNAscope labeling using *Brn3b* and *Opn4* mRNA probes in retinal sections from Vehicle- or Tamoxifen (TMX)-injected iBrn3bcKO mice. Dashed ellipses show regions of interest of ipRGC. (D) *Brn3b* mRNA levels are significantly decreased in ipRGCs from TMX-injected iBrn3bcKO (green, n=288 cells) compared to control mice (grey, n=371 cells) (P=0.0001). (E) *Brn3b* mRNA levels are significantly increased in all ipRGCs of Brn3bcKO mice (green, n=889 cells) compared to control littermates (black, n=1362 cells) (P=0.0001). (F) Proportion of *Brn3b*-negative (Brn3b-) and *Brn3b*-positive (Brn3b+) mRNA in ipRGCs from Vehicle- or TMX-injected iBrn3bcKO mice measured by RNAscope. Source data are provided as a Source Data file. Lines are median values, ****P<0.001, two-tailed Mann Whitney U test.

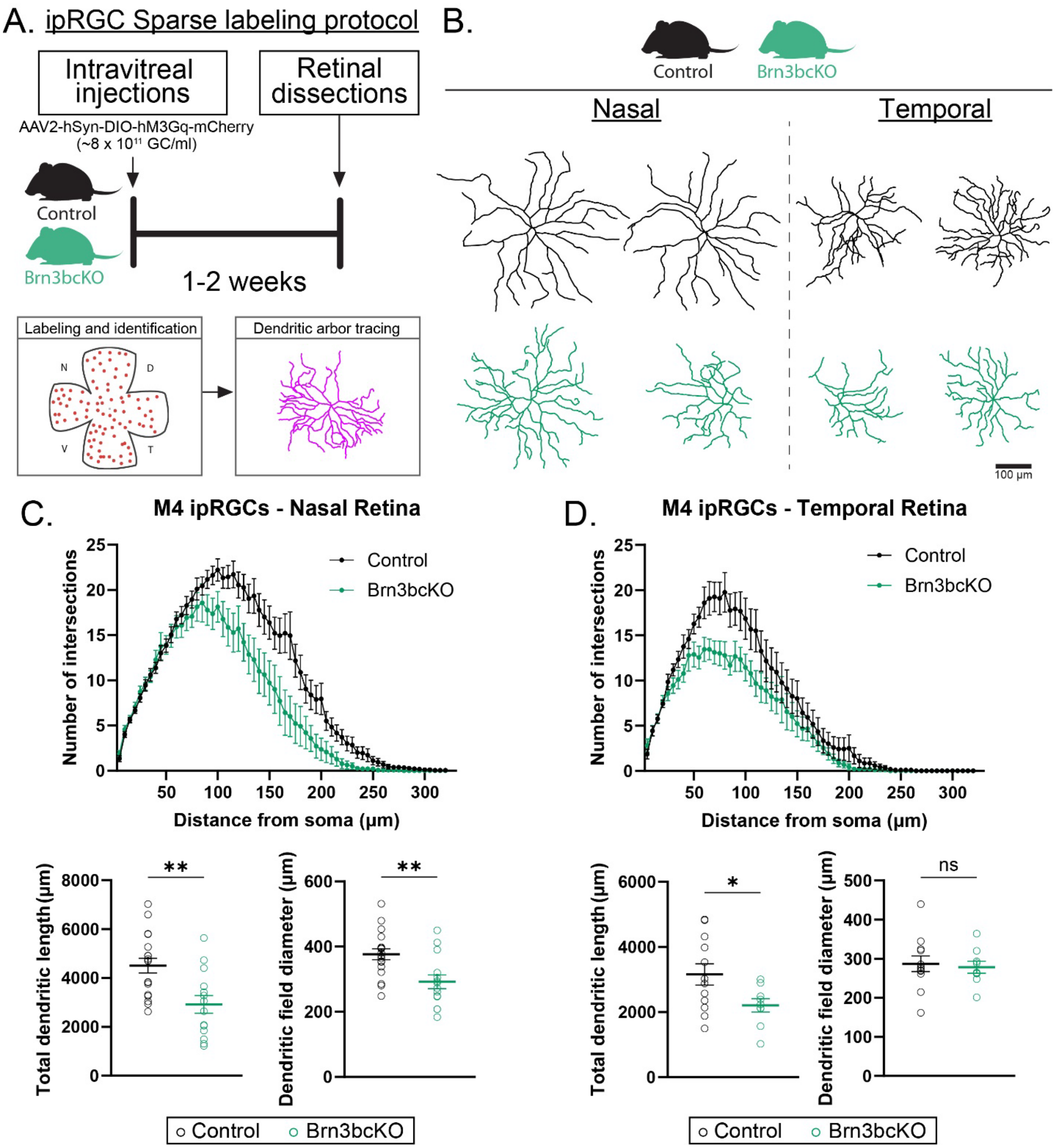

Supplementary Figure 9. Nasal M4 ipRGCs show the most pronounced changes in Brn3bcKO retinas. (A) Schematic of the ipRGC sparse labeling protocol. (B) Representative traces of nasal and temporal M4 ipRGCs in control (black) and Brn3bcKO (green) retinas. (C-D) Sholl analysis (top), total dendritic length and dendritic field diameter (bottom) from nasal (C) (n=12 cells/group;

$P=0.002$ and $P=0.003$, respectively) and temporal (D) Brn3bcKO and control retinas ($n=12$ cells in Control; $n=9$ cells in Brn3bcKO; $P=0.036$ and $P=0.741$, respectively). Source data are provided as a Source Data file. All data are Mean ± SEM, n.s. (not significant) $P>0.05$, *$P<0.05$, **$P<0.01$, two-tailed Student's t-test.

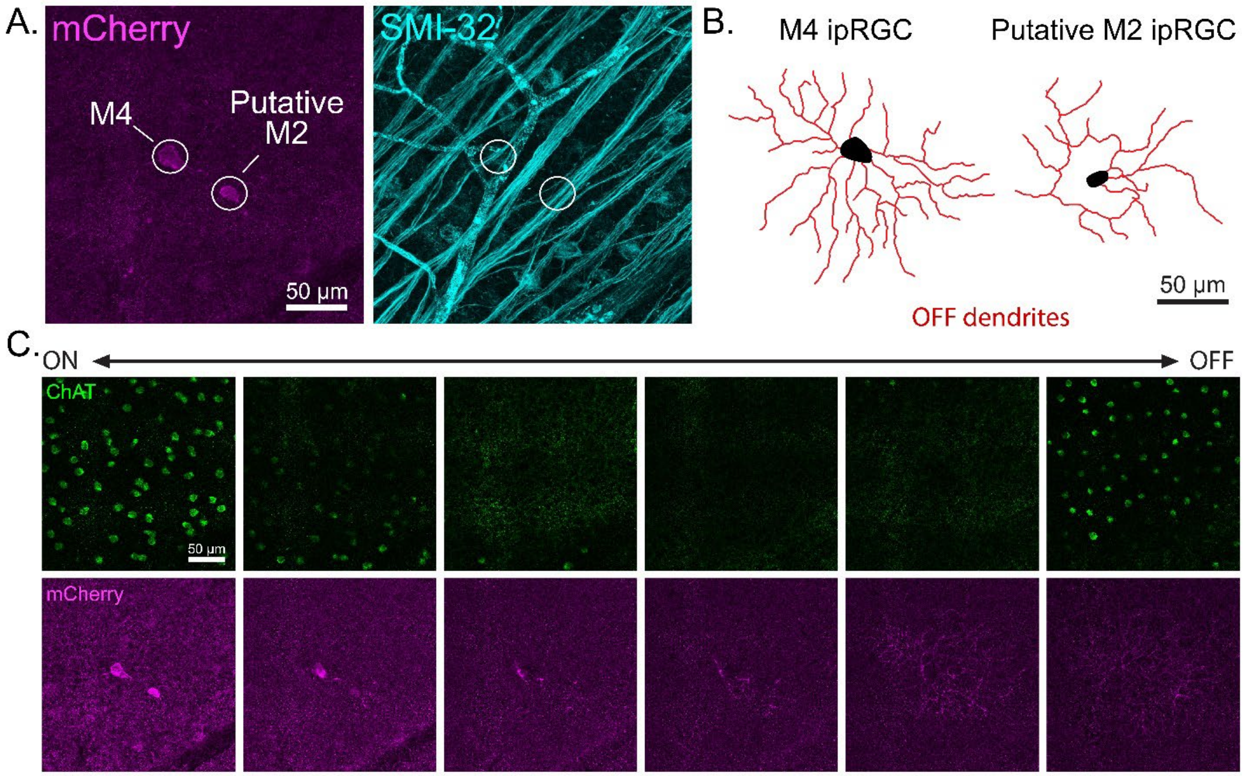

Supplementary Figure 10. Some M4 and putative M2 ipRGCs stratify dendrites in the OFF sublamina of the inner plexiform layer. (A) Representative images of spare labeled OFF-stratifying M4 ($SMI32^+$) and putative M2 ($SMI32^-$) ipRGCs in Brn3bcKO mice. (B) Traces of representative OFF-stratifying M4 and putative M2 ipRGCs in Brn3bcKO mice. (C) Stack reconstruction of M4 and putative M2 ipRGCs showing somas in ON sublayer (left) and dendritic stratification in OFF sublamina (right).

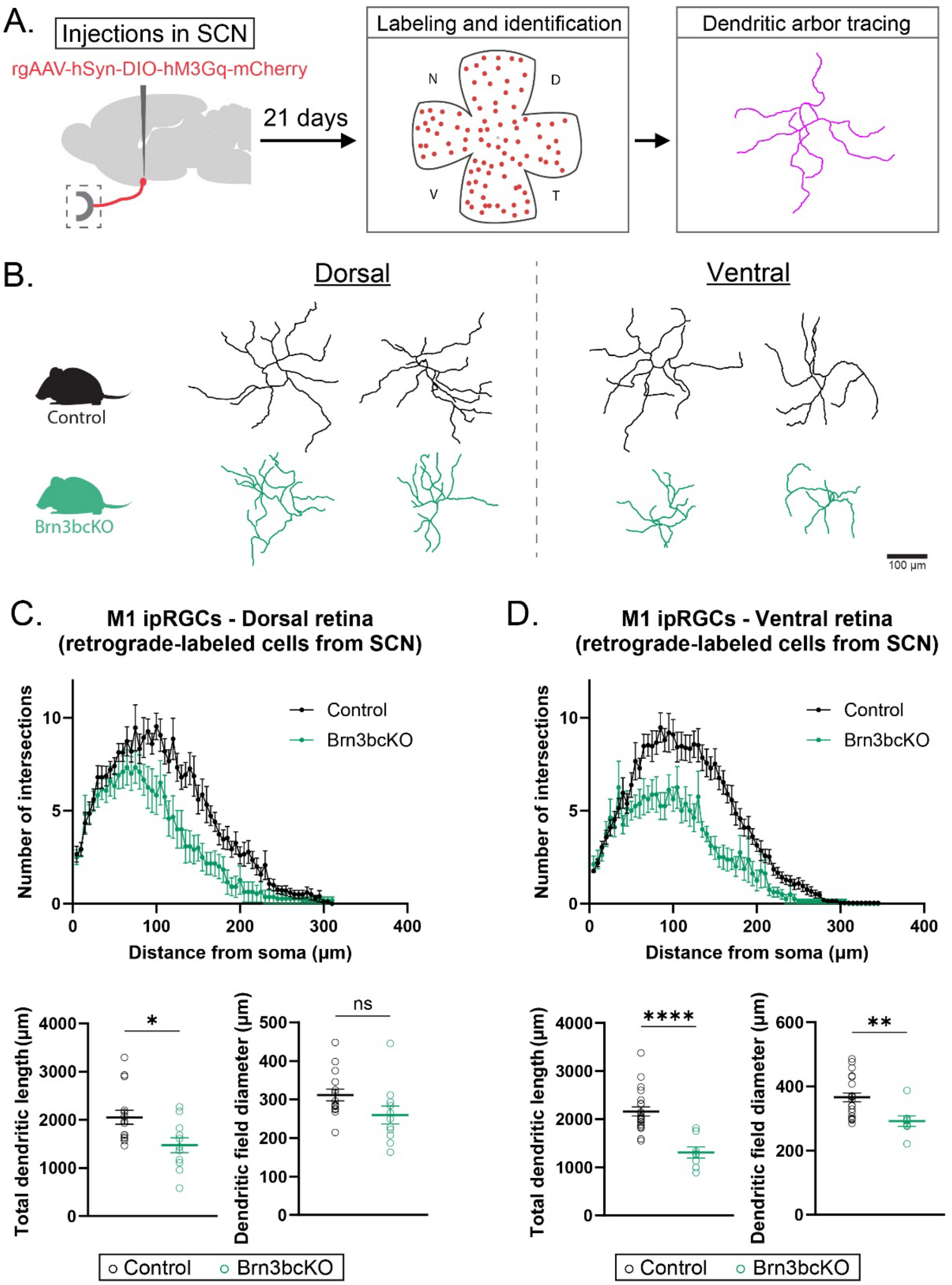

Supplementary Figure 11. Ventral M1 ipRGCs show the most pronounced changes in Brn3bcKO retinas. (A) Schematic of retrograde labeling of SCN-projecting ipRGCs and morphological study procedure. (B) Representative traces of dorsal and ventral M1 ipRGCs in control (black) and

Brn3bcKO (green) retinas. (C-D) Sholl analysis (top), total dendritic length and dendritic field diameter (bottom) from dorsal (C) (n=15 cells in Control; n=12 cells in Brn3bcKO; P=0.012 and P=0.061, respectively) and ventral (D) (n=21 cells in Control; n=8 in Brn3bcKO; P=0.0001 and P=0.005, respectively) Brn3bcKO and control retinas. Source data are provided as a Source Data file. All data are Mean ± SEM, n.s. (not significant) P>0.05, *P<0.05, **P<0.01, two-tailed Student's t-test.

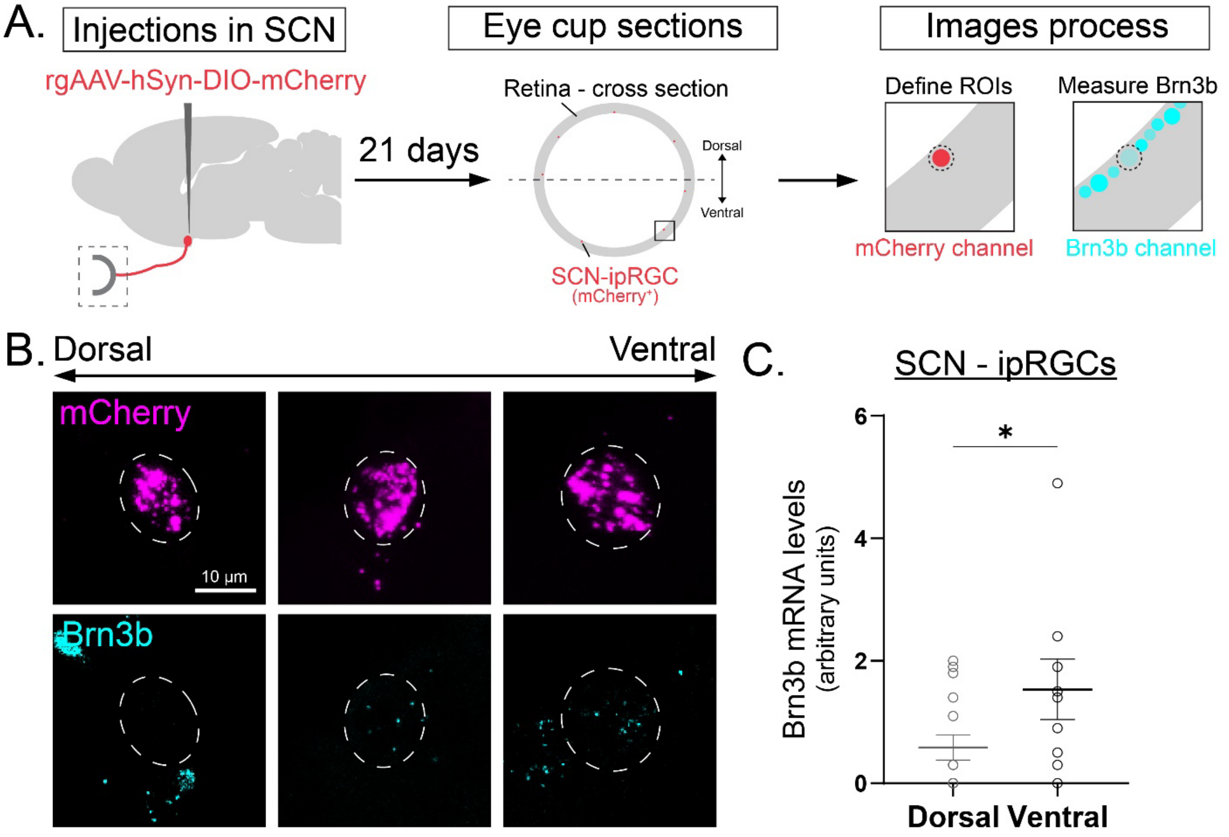

Supplementary Figure 12. Ventral SCN-M1 ipRGCs show higher levels of *Brn3b* mRNA than dorsal SCN-M1 ipRGCs. (A) Schematic of retrograde labeling of SCN-projecting ipRGCs and following RNAscope experiments. (B) Representative images of RNAscope labeling using *mCherry* and *Brn3b* mRNA probes in retinal sections from retrogradely labeled retinas from the SCN. Dashed ellipses show regions of interest of *mCherry*-positive cells (i.e. SCN-ipRGCs). (C) *Brn3b* mRNA intensity quantification in dorsal (n=15 cells) and ventral (n=9 cells) SCN-ipRGCs (P=0.0467). Source data are provided as a Source Data file. Data are Mean ± SEM, *P<0.05, two-tailed Mann Whitney U test.

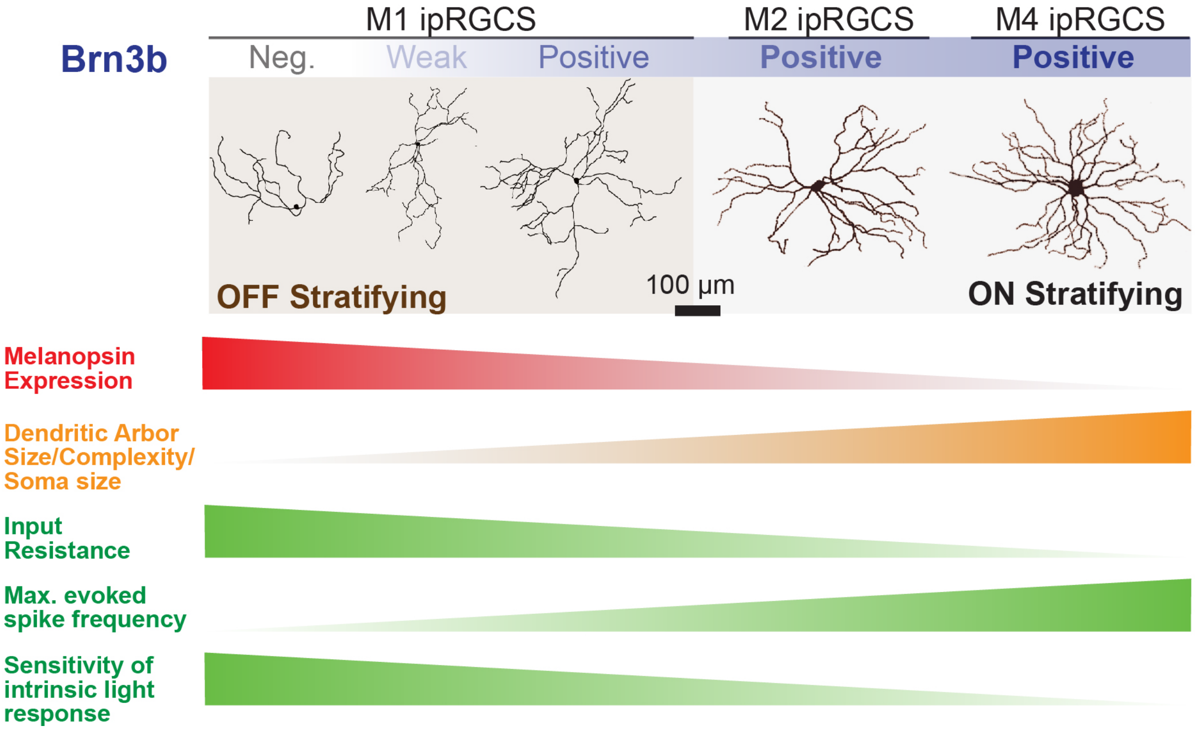

Supplementary Figure 13. *Brn3b* expression correlates with multiple features of ipRGC subtypes. Schematic representation of correlation between *Brn3b* expression levels (blue) with melanopsin expression levels (red); morphological (orange) and physiological properties (green).

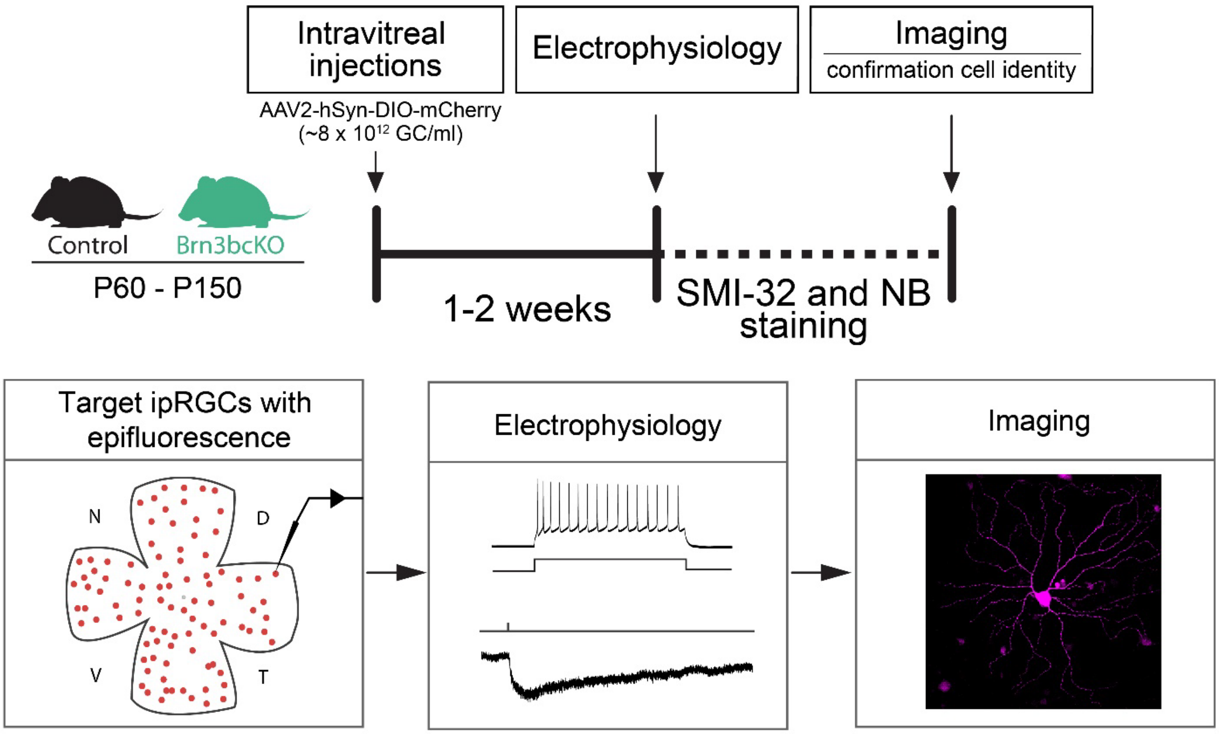

Supplementary Figure 14. Schematic of electrophysiological recordings of M4 ipRGCs from control and Brn3bcKO mice. Timeline of intravitreal injections to label ipRGCs, electrophysiological experiments and *post hoc* imaging to confirm M4 ipRGC cell identity.

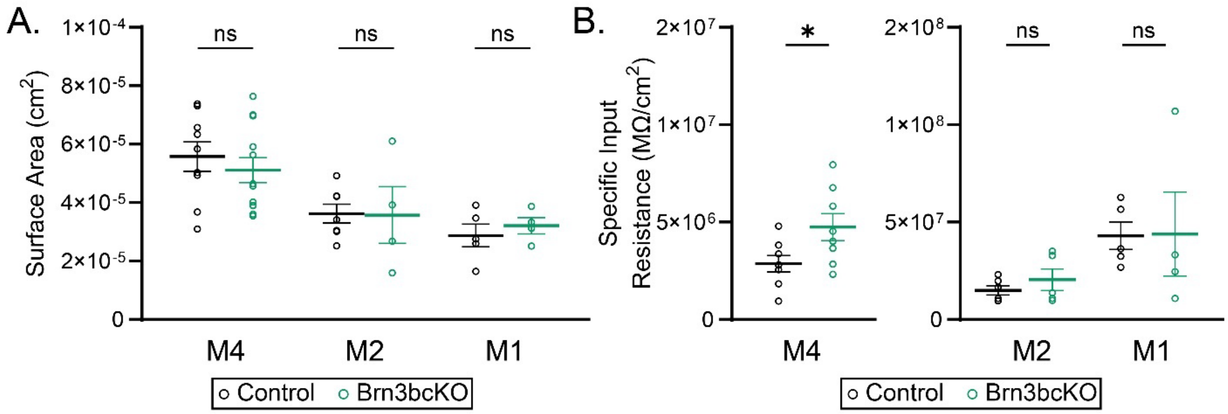

Supplementary Figure 15. Capacitance and specific input in Brn3bcKO and Control ipRGC subtypes. (A) No differences observed in surface area in recorded Brn3bckO (green) and Control (black) ipRGC subtypes (n=9 in Control M4, n=8 in Brn3bcKO M4, P=0.488; n=5 in Control M2, n=4 in Brn3bcKO M2, P=0.953; n=5 in Control M1, n=4 in Brn3bcKO M1, P=0.530). (B) Brn3bcKO M4 ipRGC presented a higher specific input while no differences were observed in M2 and M1 ipRGCs (n=9 in Control M4, n=8 in Brn3bcKO M4, P=0.036; n=5 in Control M2, n=4 in Brn3bcKO M2, P=0.358; n=5 in Control M1, n=4 in Brn3bcKO M1, P=0.556). Source data are provided as a Source Data file. Data are Mean ± SEM, n.s. (not significant) when P>0.05, *P<0.05, two-tailed Student's t- and Mann Whitney U tests.

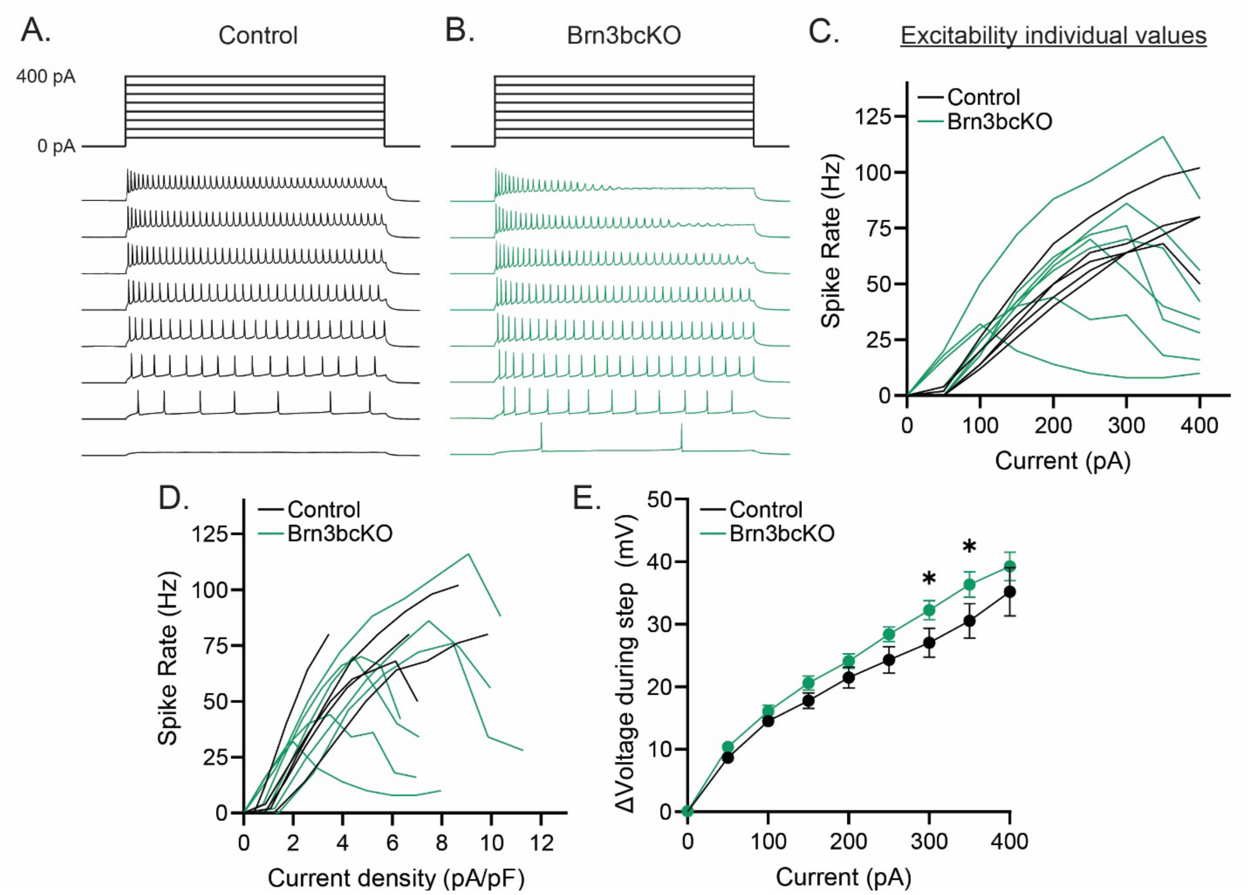

Supplementary Figure 16. BRN3B tunes the excitability of M4 ipRGCs. (A-B) Individual traces of evoked firing response to current injections in control (A, black) and Brn3bcKO (B, green) M4 ipRGCs. (C-D) Individual traces of evoked firing response at different current steps (C) and current density (D) recorded in control (black, n=5) and Brn3bcKO (green, n=7) M4 ipRGCs. (E) Difference of voltage between baseline and after the current injection step control (black, n=5) and Brn3bcKO (green, n=7) in M4 ipRGCs (300 pA: P=0.031; 350 pA: P=0.016). Source data are provided as a Source Data file. Data are Mean ± SEM, *P<0.05, two-way ANOVA with repeated measures.

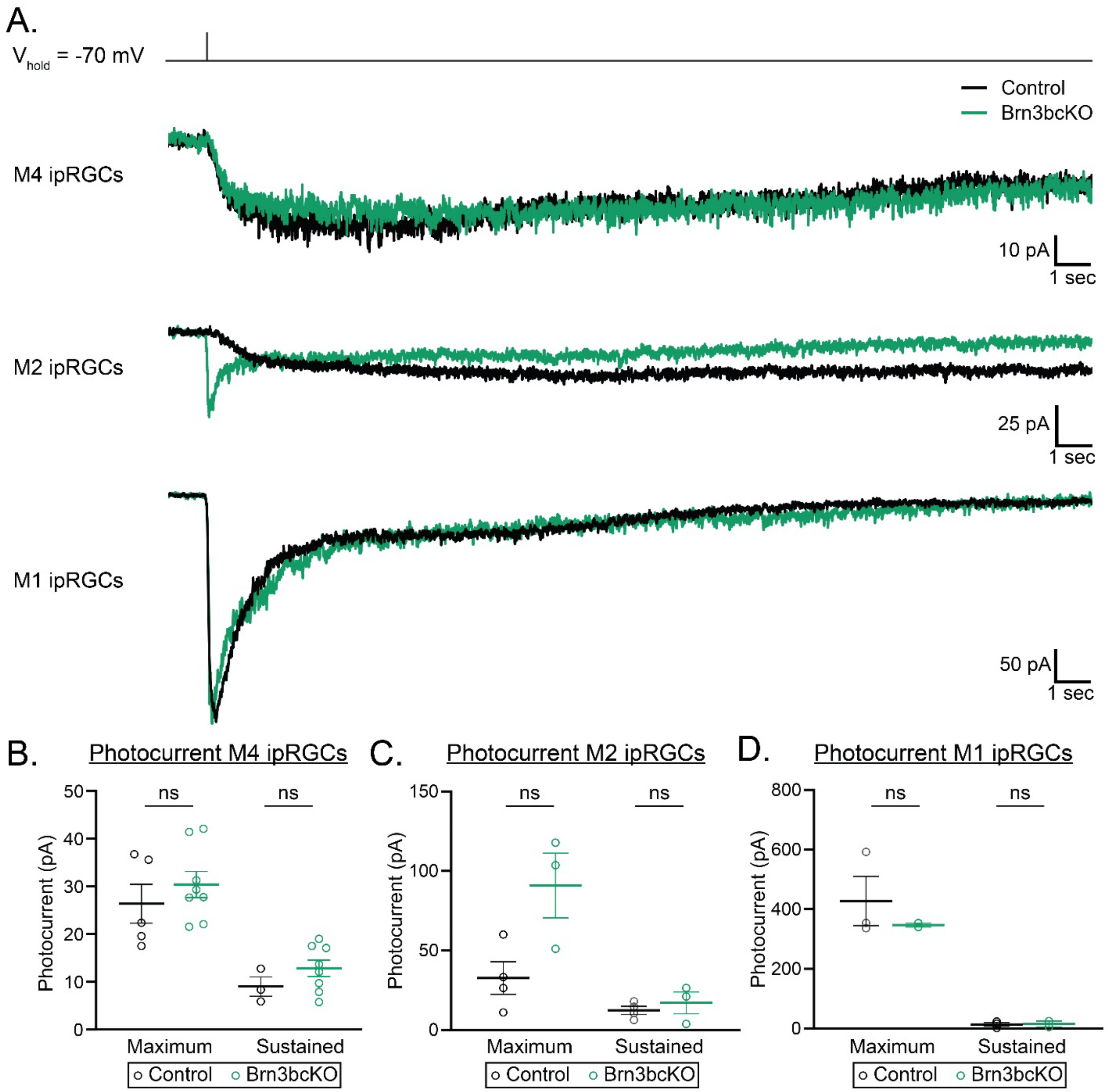

Supplementary Figure 17. BRN3B does not affect the intrinsic photocurrent of M4, M2 and M1 ipRGCs. (A) Representative traces of photocurrent responses in control (black), Brn3bcKO (green) of M4 (top), M2 (middle) and M1 (bottom) ipRGCs. (B-D) Maximum and sustained photocurrent control and Brn3bcKO M4 (B) (n=5 in Control, n=8 in Brn3bcKO, Maximum: P=0.411, Sustained: P=0.376), M2 (C) (n=4 in Control, n=3 in Brn3bcKO, Maximum: P=0.114, Sustained: P=0.496) and M1 (D) (n=3 in Control, n=2 in Brn3bcKO, Maximum: P=0.800, Sustained:

P=0.936) ipRGCs. Source data are provided as a Source Data file. Data are Mean ± SEM, n.s. (not significant), P>0.05, two-tailed Mann Whitney U test.

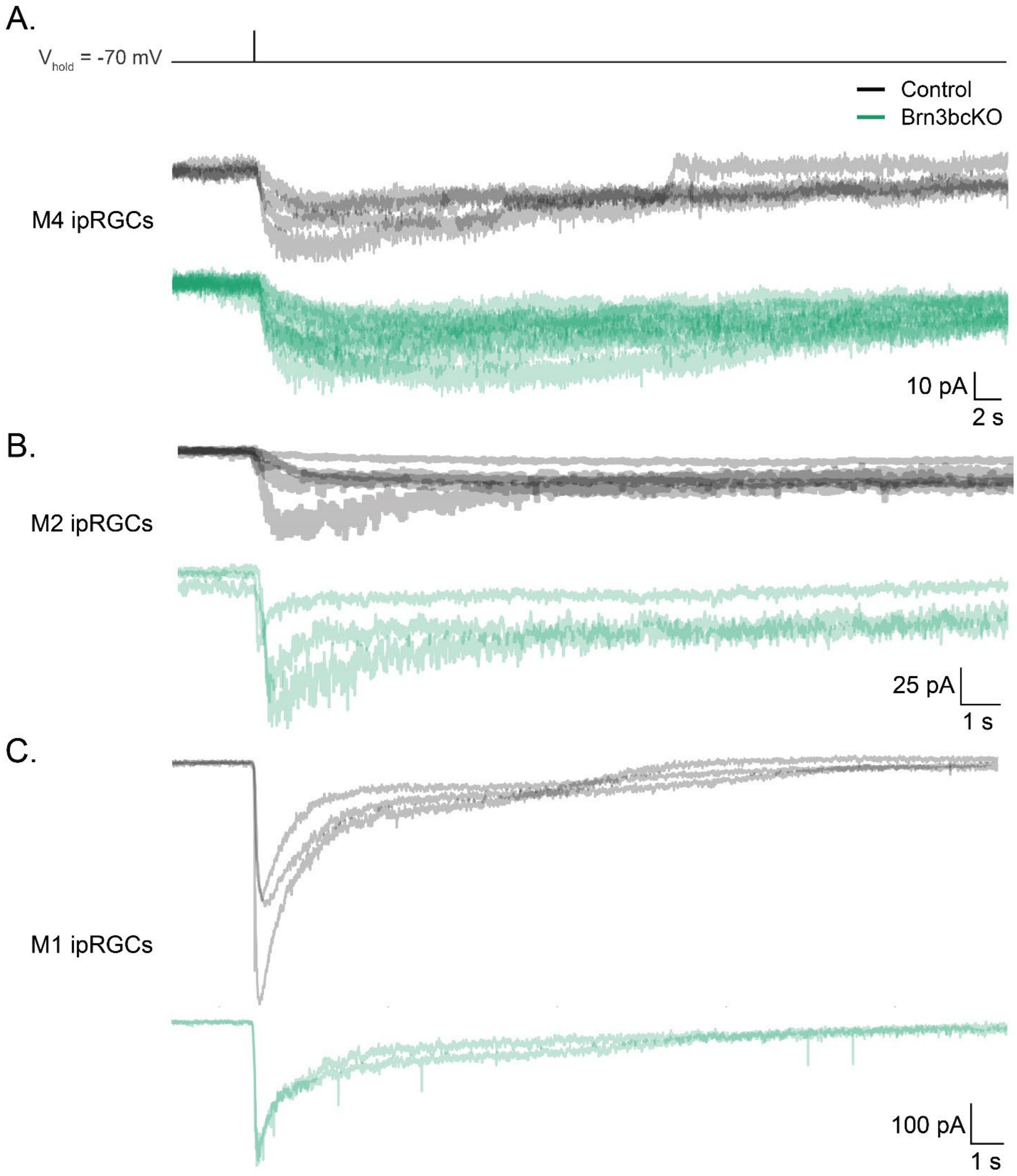

Supplementary Figure 18. Photocurrent raw traces of control and Brn3bcKO ipRGCs. (A-C) Raw traces of photocurrent responses in control (black), Brn3bcKO (green) of M4 (A), M2 (B) and M1 (C) ipRGCs.

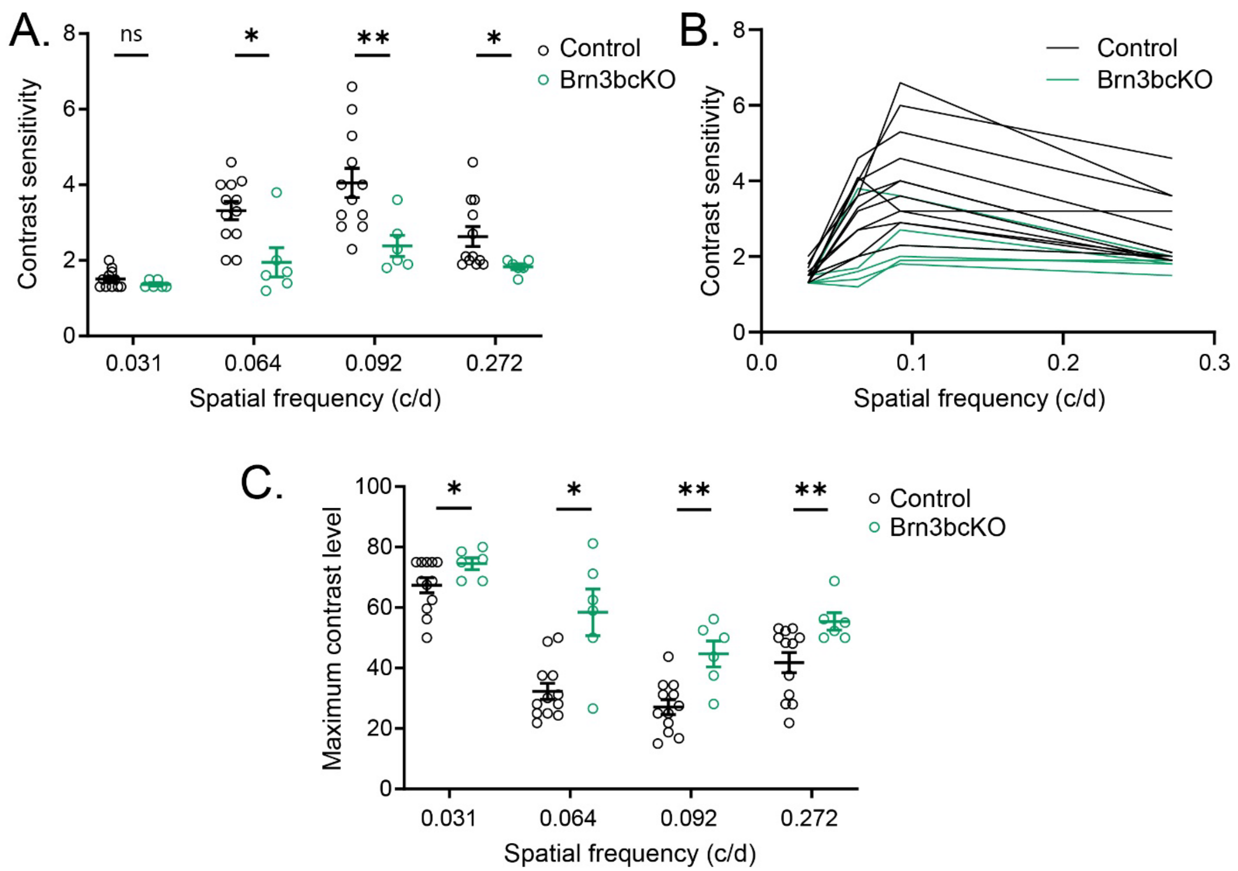

Supplementary Figure 19. Brn3bcKO mice showed decreased contrast sensitivity. (A) contrast sensitivity quantification in OKR at 0.031, 0.064, 0092 and 0.272 cycles/degree (c/d) in control (black, n=12) and Brn3bcKO (green, n=6) mice (0.031 cpd, P=0.176; 0.064 cpd, P=0.006; 0.092 cpd, P=0.011; 0.272, P=0.128). (B) individual traces of contrast sensitivity vs. spatial frequencies in control (black, n=12) and Brn3bcKO (green, n=6) mice. (C) individual values of maximum contrast level vs. spatial frequencies in control (black, n=12) and Brn3bcKO (green, n=6) mice (0.031 cpd, P=0.376; 0.064 cpd, P=0.018; 0.092 cpd, P=0.007; 0.272, P=0.008). Source data are provided as a Source Data file. Data are Mean ± SEM, n.s. (not significant) P>0.05, *P<0.05, **P<0.01, two-way ANOVA with repeated measures.

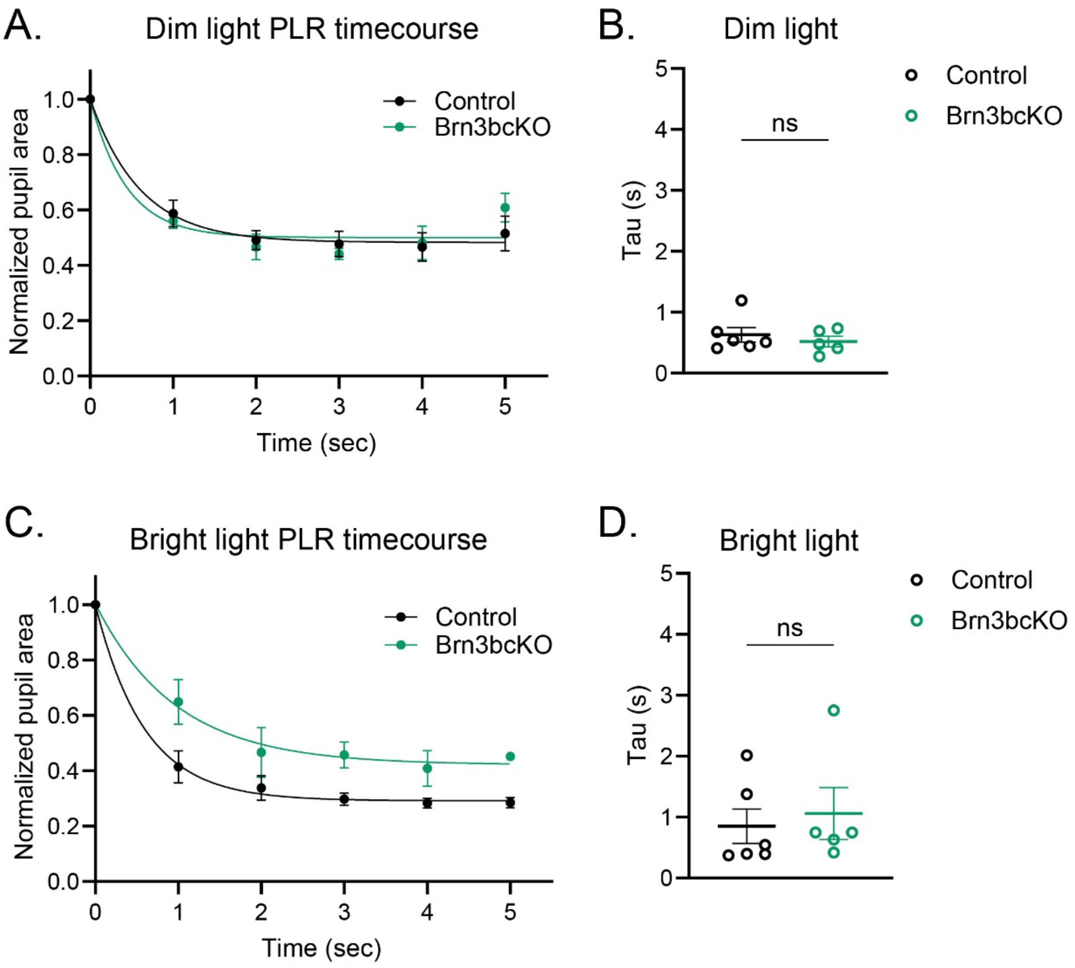

Supplementary Figure 20. Time course of pupillary light response (PLR) in control and Brn3bcKO mice. (A and C) Pupil constriction plotted as a function of time in control (black) and Brn3bcKO (green) mice in response to 5 second dim (13.8 log quanta/cm2/s, A) and bright (14.8 log quanta/cm2/s, C) light stimuli. (B and D) Grouped data of the time constant Tau measured by fitting individual PLR data using a single-exponential decay function. There were no significant differences in Tau between control (black, n=6) and Brn3bKO (green, n=5) animals in response to dim (B) (P=0.329) and bright (D) (P=0.792) light stimuli. Source data are provided as a Source Data file. Data are Mean ± SEM, n.s. (not significant) P>0.05, two-tailed Mann-Whitney U test.

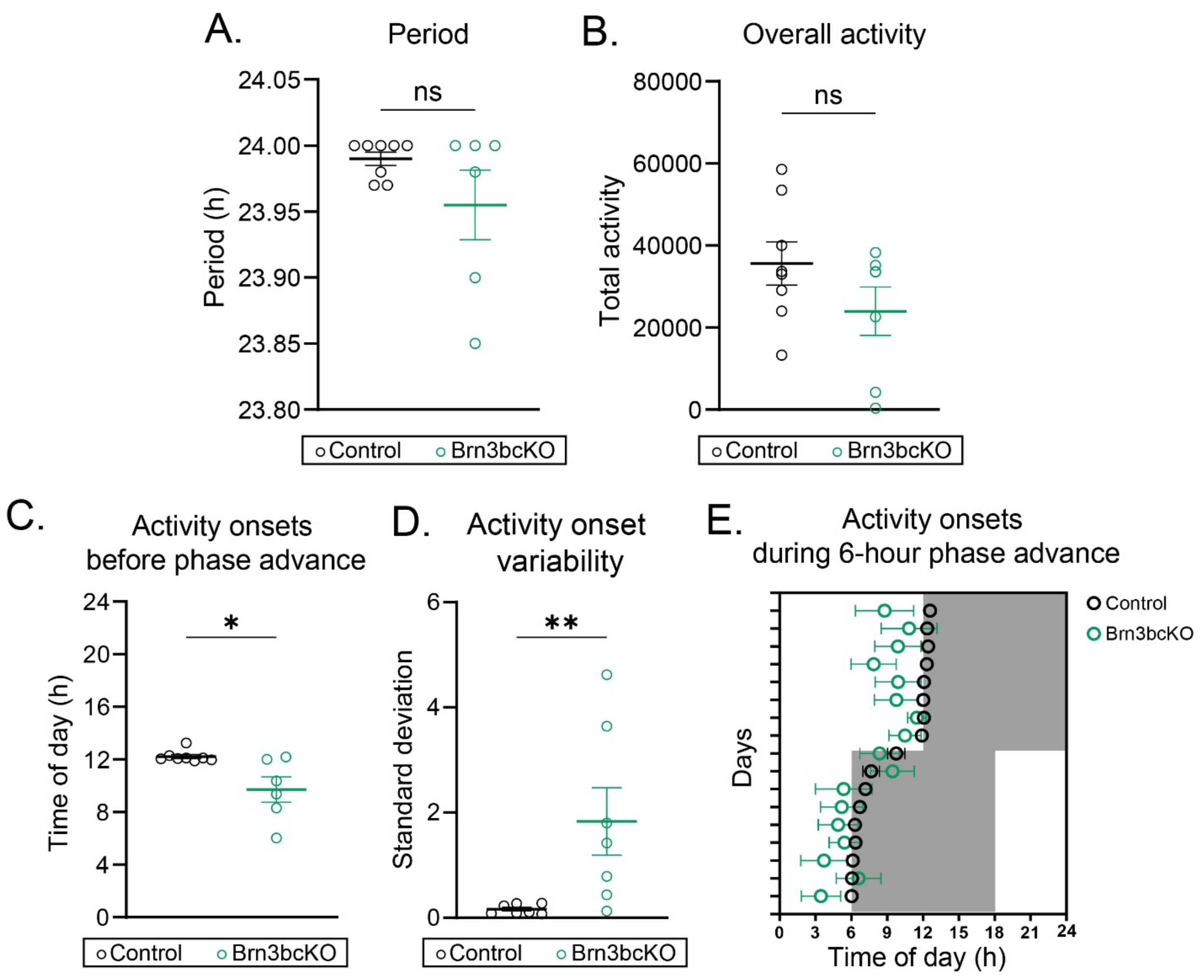

Supplementary Figure 21. Period and activity profile parameters in control and Brn3bcKO. (A-B) Brn3bcKO (green) did not show different period length (A) or overall activity changes (B) compared to control (black) mice (n = 6 / group, P=0.157, P=0.163, respectively). (C-D) The average activity onset was earlier (C) and more variable (D) in Brn3bcKO (green) mice compared to control (black) (n = 6 / group, P=0.011, P=0.004, respectively). (E) The activity onsets during 6-hour phase advance were not different in Brn3bcKO mice (n = 6 / group). Source data are provided as a Source Data file. Data are Mean ± SEM, n.s. (not significant) P>0.05, *P<0,05, **P<0.01, two-tailed Student's t and Mann Whitney U tests.

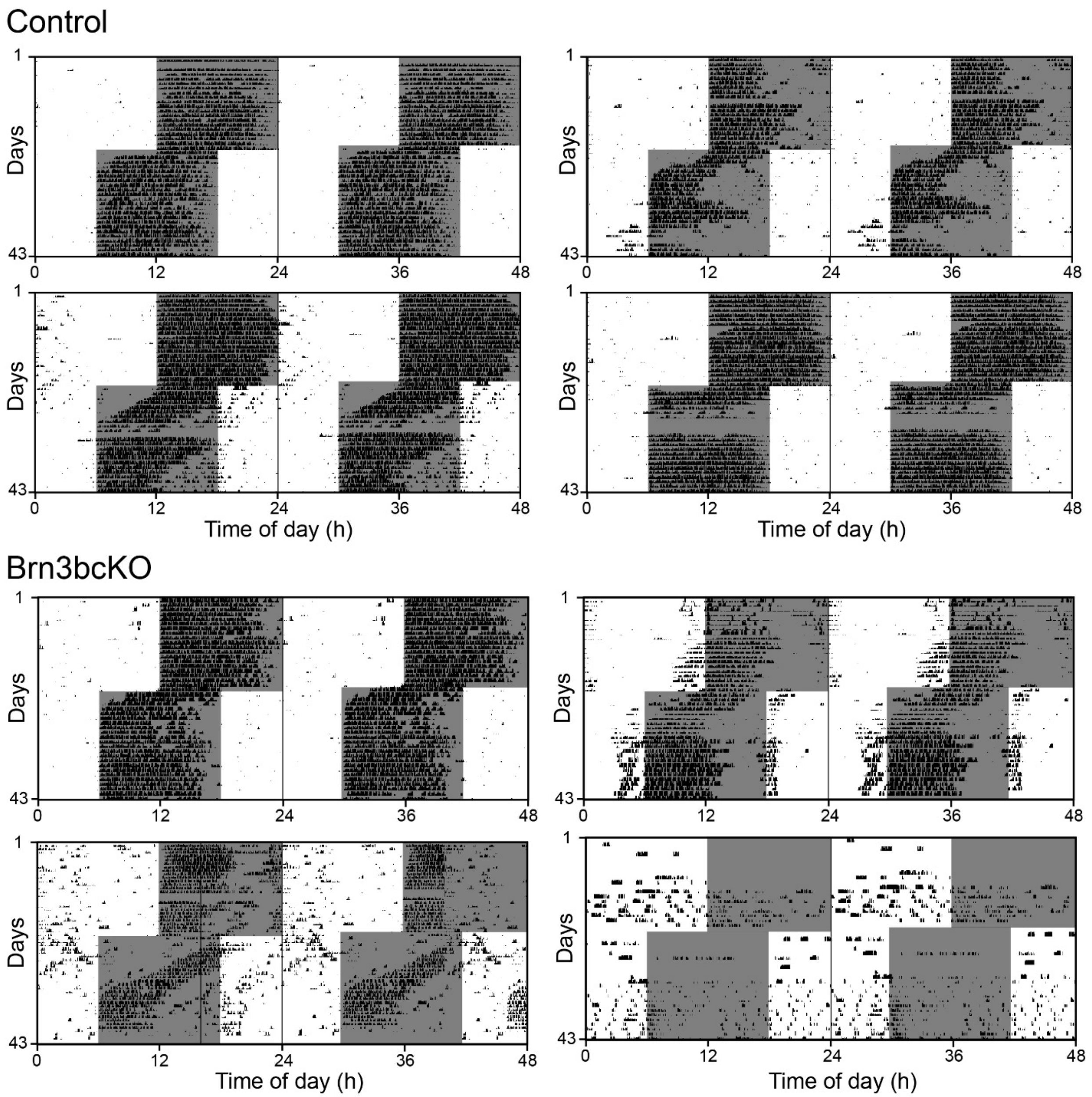

Supplementary Figure 22. Representative actograms from control and Brn3bcKO mice. Representative wheel running activity profiles from control (top) and Brn3bcKO (bottom) mice.

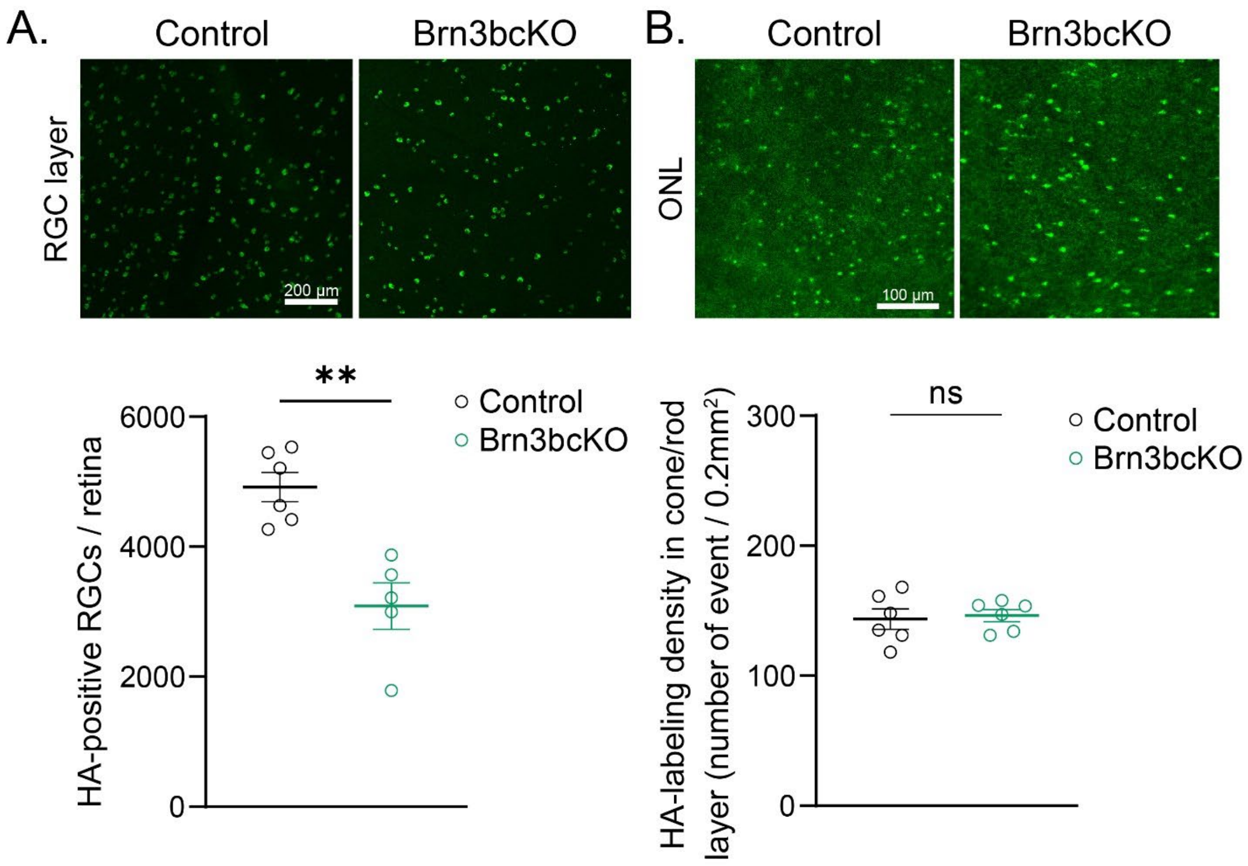

Supplementary Figure 23. Characterization of $Opn4^{Cre/+}$; $Rpl22^{HA}$ and $Opn4^{Cre/+}$; $Brn3b^{cKOAP}$ $Rpl22^{HA}$ mouse lines. (A) (Top) HA expression in the retinal ganglion cell (RGC) layer in control and Brn3bcKO mice. (Bottom) Brn3bcKO (green, n=5 retinas) mice presented significantly lower number of HA-expressing RGC compared to control (black, n=6 retinas) littermates (P=0.001). (B) (Top) HA expression in the outer nuclear layer (ONL) in control and Brn3bcKO mice. (Bottom) control (black, n=6 retinas) and Brn3bcKO (green, n=5 retinas) mice presented non-significant differences in the density of cone photoreceptors expressing HA (P=0.765). Source data are provided as a Source Data file. All data are Mean ± SEM, n.s. (not significant) P>0.05, **P<0.01, two-tailed Mann Whitney U test.

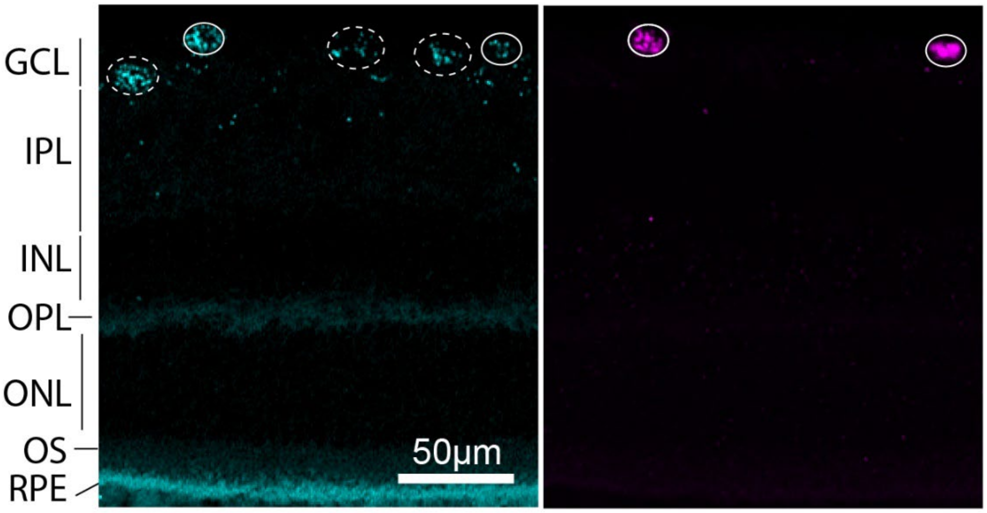

Supplementary Figure 24. Pattern labeling of *Brn3b* and *Opn4* mRNA in cross-sectioned retinas. Brn3b mRNA probe (left, cyan) is present in the retinal ganglion cell layer (GCL) in ipRGCs (full ellipse), other RGCs (dashed line ellipse) and sparse puncta in the inner plexiform layer (IPL). *Opn4* mRNA probe (Right, magenta) is present only in the GCL in ipRGCs (full ellipse). INL: inner nuclear layer; OPL: outer plexiform layer; ONL: outer nuclear layer; OS: outer segments of the cone and rod photoreceptors; RPE: retinal pigmented epithelium.

Probe	Catalog number	Tissue	Fluorophore
Mm-*Rbpms*	527231	Retinal sections	Fluorescein (NEL741001KT)
Mm-*Opn4*-C2	438061-C2	Retinal sections	Cyanine 3 (NEL744001KT)
Mm-*Brn3b*-C3 (Mm-*Pou4f2*-C3)	558481-C3	Retinal sections	Cyanine 5 (NEL745001KT)
Mm-*Opn4*	438061	Retinal sections	Cyanine 3 (NEL744001KT)
Mm-*Eomes*-C2	429641-C2	Retinal sections	Fluorescein (NEL741001KT)
Mm-*mCherry*-C2	431201-C2	Retinal sections	Cyanine 3 (NEL744001KT)
Mm-*Opn4*	438061	Whole mount retinas	Fluorescein (NEL741001KT)
Mm-*Chrna6*-C2	467711-C2	Whole mount retinas	Cyanine 3 (NEL744001KT)
Mm-*Zcchc12*-C3	524441-C3	Whole mount retinas	Cyanine 5 (NEL745001KT)

Supplementary Table 1. List of mRNA probes and fluorophores used in this study. All fluorophores were used 1:1000.

Purpose	Primary Ab / Tracer	Dilution	Secondary Ab
To characterize $Opn4^{Cre/+}$ $Rpl22^{HA}$ and $Opn4^{Cre/+}$; $Brn3b^{cKOAP}$ $Rpl22^{HA}$ lines (Supplementary Figure 23)	Rabbit anti-HA (Abcam, ab9110)	1:500	Donkey anti-rabbit Alexa 488 (Thermofisher, A-21206)
To test the role of Brn3b on melanopsin expression (Fig. 2, Exteded Data Figs. 5 & 7)	Rabbit anti-melanopsin (ATS, N38)	1:1000	Donkey anti-rabbit Alexa 488 (Thermofisher, A-21206)
	Rabbit anti-Calbindin (Swant, CB-38a)	1:500	
Morphological studies (sparse labeling) (Fig. 3, Extended Data Figs. 9 & 10)	Rabbit dsRed (Takara, 632496)	1:500	Donkey anti-rabbit Alexa 594 (ThermoFisher, A-21207)
	Goat anti-ChAT (Sigma, AB144P)	1:250	Donkey anti-goat Alexa 488 (Invitrogen, A11055)
	Mouse anti-SMI32 (Biolegend, 801701)	1:500	Donkey anti-mouse Alexa 647 (Invitrogen, A31571)
Morphological studies (filled cells) (Fig. 3, Extended Data Figs. 11 & 12)	Neurobiotin (VectorLabs, SP-1120-50)	-	Streptavidin 546 (Invitrogen, S11225)
	Chicken anti-GFP (Abcam, ab13970)	1:500	Donkey anti-chicken Alexa 488 (Jackson IRL, 703-545-155)
	Goat anti-Brn3b (Abcam, ab56026)	1:250	Donkey anti-goat Alexa 647 (Invitrogen, A21447)
Electrophysiological studies (filled cells) (Fig. 4; Extended Data Figs 14-18)	Neurobiotin (VectorLabs, SP-1120-50)	-	Streptavidin 546 (Invitrogen, S11225)
	Mouse anti-SMI32 (Biolegend, 801701)	1:500	Donkey anti-mouse Alexa 488 (Abcam, ab150105)

Supplementary Table 2. List of antibodies and tracers used in this study. The dilution of all secondary antibodies and streptavidin was 1:500. All antibodies used in this study have been

previously validated by the manufacturers. Validation data and protocols are available on the respective manufacturers' websites.
