# Supplementary material for: Genetic tuning of retinal ganglion cell subtype identity to drive visual behavior
Source: Nat Commun. 2025 Sep 30;16:8678. doi: 10.1038/s41467-025-63675-w (PMC12484735; doi:10.1038/s41467-025-63675-w)
Supplement: Supplementary file 2 — Description of Additional Supplementary Files [file 41467_2025_63675_MOESM2_ESM.pdf]

Description of Additional Supplementary Files

Supplementary Data 1: Differentially expressed transcripts in ipRGCs upon knockout of BRN3B using TRAPseq (two-sided P-value from negative binomial distribution with Benjamini-Hochberg post-hoc test, false discovery rate (FDR)<0.05)

Supplementary Data 2: BRN3B direct target genes with shared expression between E14.5 retina[21] and adult ipRGCs[3] .
