# Supplementary material for: Genetic tuning of retinal ganglion cell subtype identity to drive visual behavior
Source: Nat Commun. 2025 Sep 30;16:8678. doi: 10.1038/s41467-025-63675-w (PMC12484735; doi:10.1038/s41467-025-63675-w)
Supplement: Supplementary file 5 — Reporting Summary [file 41467_2025_63675_MOESM5_ESM.pdf]

nature portfolio

Corresponding author(s): Dr. Tiffany M. Schmidt

Last updated by author(s): Jun 24, 2025

Reporting Summary

Nature Portfolio wishes to improve the reproducibility of the work that we publish. This form provides structure for consistency and transparency in reporting. For further information on Nature Portfolio policies, see our Editorial Policies and the Editorial Policy Checklist.

Statistics

For all statistical analyses, confirm that the following items are present in the figure legend, table legend, main text, or Methods section.

n/a	Confirmed	
☐	☒	The exact sample size (*n*) for each experimental group/condition, given as a discrete number and unit of measurement
☐	☒	A statement on whether measurements were taken from distinct samples or whether the same sample was measured repeatedly
☐	☒	The statistical test(s) used AND whether they are one- or two-sided<br>*Only common tests should be described solely by name; describe more complex techniques in the Methods section.*
☐	☒	A description of all covariates tested
☐	☒	A description of any assumptions or corrections, such as tests of normality and adjustment for multiple comparisons
☐	☒	A full description of the statistical parameters including central tendency (e.g. means) or other basic estimates (e.g. regression coefficient) AND variation (e.g. standard deviation) or associated estimates of uncertainty (e.g. confidence intervals)
☐	☒	For null hypothesis testing, the test statistic (e.g. *F*, *t*, *r*) with confidence intervals, effect sizes, degrees of freedom and *P* value noted<br>*Give P values as exact values whenever suitable.*
☒	☐	For Bayesian analysis, information on the choice of priors and Markov chain Monte Carlo settings
☒	☐	For hierarchical and complex designs, identification of the appropriate level for tests and full reporting of outcomes
☒	☐	Estimates of effect sizes (e.g. Cohen's *d*, Pearson's *r*), indicating how they were calculated

Our web collection on statistics for biologists contains articles on many of the points above.

Software and code

Policy information about availability of computer code

Data collection	• pClamp 10 (RRID:SCR_011323) – Used for acquisition of electrophysiology recordings via the Multiclamp 700B amplifier.<br>• ClockLab Data Collection software (Actimetrics) – Used to record wheel-running activity for circadian rhythm analysis.<br>• OptoMotry system (Cerebral Mechanics) – Used for quantifying visual behavior via optokinetic tracking response.<br>• Sony Handycam camcorder – Used to record pupillary light reflex (PLR) responses.<br>• Leica SPE5500 confocal microscope – Used to acquire fluorescence images of retinal sections and whole-mounts.<br>• Leica CM1950 cryostat – Used for sectioning tissue for RNA FISH and immunohistochemistry.<br>• Qubit fluorometer (Thermo Fisher Scientific) and Agilent Bioanalyzer – Used for quantification and quality control of RNA libraries.<br>• Illumina NextSeq 550 – Used for sequencing Smart-seq3 RNA-seq libraries.
Data analysis	• GraphPad Prism v10.1.2 (RRID:SCR_002798) – Used for statistical analyses and generation of plots.<br>• ImageJ (RRID:SCR_003070) – Used for quantifying pixel intensity, drawing regions of interest (ROIs), and normalizing image data.<br>• Seurat v4 (RRID:SCR_016341) – Used for scRNA-seq data analysis, integration, dimensionality reduction (UMAP), clustering, and module score calculations.<br>• edgeR (RRID:SCR_012802) – Used for differential gene expression analysis of TRAP-seq data.<br>• Smart-seq3 pipeline – GitHub pipeline used to align and quantify Smart-seq3 libraries: https://github.com/sandberg-lab/Smart-seq3.<br>• fastMNN (from the batchelor Bioconductor package) – Used for batch correction and integration of scRNA-seq datasets.<br>• Simple Neurite Tracer (SNT) – Used in ImageJ for quantification of dendritic arbors; available at https://github.com/morphonets/SNT.<br>• PyABF – Python package used to analyze electrophysiology data; https://pypi.org/project/pyabf/.<br>• Python Programming Language v3.12.7 (RRID: SCR_008394) - Used to analyze electrophysiology data<br>• Custom Python scripts – Developed for analyzing ipRGC electrophysiological recordings; available at https://github.com/schmidtlab-northwestern.

- DeepLabCut (RRID:SCR_017302) – Used to extract pupil diameter over time from video recordings of PLR responses.
- ClockLab Analysis Software v6 (Actimetrics) – Used for circadian rhythm data analysis from wheel-running behavior.
- Matlab (RRID:SCR_001622) – Used for additional custom data analysis.
- R (RRID:SCR_001905) – Used for additional custom data analysis.

For manuscripts utilizing custom algorithms or software that are central to the research but not yet described in published literature, software must be made available to editors and reviewers. We strongly encourage code deposition in a community repository (e.g. GitHub). See the Nature Portfolio guidelines for submitting code & software for further information.

Data

Policy information about availability of data

All manuscripts must include a data availability statement. This statement should provide the following information, where applicable:

- Accession codes, unique identifiers, or web links for publicly available datasets
- A description of any restrictions on data availability
- For clinical datasets or third party data, please ensure that the statement adheres to our policy

Source Data are provided with this paper. TRAP-seq data are available in the Gene Expression Omnibus (GEO) database under the reference number GSE274888 [https://www.ncbi.nlm.nih.gov/geo/query/acc.cgi?acc=GSE274888]. Mouse RGC snRNAseq and BRN3B CUT&Tag data were downloaded through the GEO repository under the accession codes: GSE137400 [https://www.ncbi.nlm.nih.gov/geo/query/acc.cgi?acc=GSE137400] and GSE220587 [https://www.ncbi.nlm.nih.gov/geo/query/acc.cgi?acc=GSE220587].

Research involving human participants, their data, or biological material

Policy information about studies with human participants or human data. See also policy information about sex, gender (identity/presentation), and sexual orientation and race, ethnicity and racism.

Reporting on sex and gender	n/a
Reporting on race, ethnicity, or other socially relevant groupings	n/a
Population characteristics	n/a
Recruitment	n/a
Ethics oversight	n/a

Note that full information on the approval of the study protocol must also be provided in the manuscript.

Field-specific reporting

Please select the one below that is the best fit for your research. If you are not sure, read the appropriate sections before making your selection.

☒ Life sciences ☐ Behavioural & social sciences ☐ Ecological, evolutionary & environmental sciences

For a reference copy of the document with all sections, see nature.com/documents/nr-reporting-summary-flat.pdf

Life sciences study design

All studies must disclose on these points even when the disclosure is negative.

Sample size	Sample sizes were chosen to be commensurate with previous studies examining similar properties and are noted in the figure legends.
Data exclusions	No data were excluded from the analyses
Replication	4 biological replicates were used for sequencing experiments. We confirm all replication attempts were successful.
Randomization	Samples were allocated into experimental groups based on animal genotype. All experiments included littermate controls.
Blinding	All measurements and analyses were automated and all experimental groups were subjected to the same analysis protocols regardless of genotype.

Reporting for specific materials, systems and methods

We require information from authors about some types of materials, experimental systems and methods used in many studies. Here, indicate whether each material, system or method listed is relevant to your study. If you are not sure if a list item applies to your research, read the appropriate section before selecting a response.

Materials & experimental systems

n/a	Involved in the study
☐	☒ Antibodies
☒	☐ Eukaryotic cell lines
☒	☐ Palaeontology and archaeology
☐	☒ Animals and other organisms
☒	☐ Clinical data
☒	☐ Dual use research of concern
☒	☐ Plants

Methods

n/a	Involved in the study
☒	☐ ChIP-seq
☒	☐ Flow cytometry
☒	☐ MRI-based neuroimaging

Antibodies

Antibodies used

Primary Antibodies / Tracers
- Rabbit anti-HA (Abcam, ab9110), 1:500
- Rabbit anti-melanopsin (ATS, N38), 1:1000
- Rabbit anti-Calbindin (Swant, CB-38a), 1:500
- Rabbit dsRed (Takara, 632496), 1:500
- Goat anti-ChAT (Sigma, AB144P), 1:250
- Mouse anti-SMI32 (Biolegend, 801701), 1:500
- Neurobiotin (VectorLabs, SP-1120-50), -
- Chicken anti-GFP (Abcam, ab13970), 1:500
- Goat anti-Brn3b (Abcam, ab56026), 1:250
- Mouse anti-SMI32 (Biolegend, 801701), 1:500

Secondary Antibodies
- Donkey anti-rabbit Alexa 488 (ThermoFisher, A-21206)
- Donkey anti-rabbit Alexa 488 (ThermoFisher, A-21206)
- Not specified
- Donkey anti-rabbit Alexa 594 (ThermoFisher, A-21207)
- Donkey anti-goat Alexa 488 (Invitrogen, A11055)
- Donkey anti-mouse Alexa 647 (Invitrogen, A31571)
- Streptavidin 546 (Invitrogen, S11225)
- Donkey anti-chicken Alexa 488 (Jackson IRL, 703-545-155)
- Donkey anti-goat Alexa 647 (Invitrogen, A21447)
- Donkey anti-mouse Alexa 488 (Abcam, ab150105)

Validation

All antibodies used in this study have been previously validated by the manufacturers. Validation data and protocols are available on the respective manufacturers' websites.

Animals and other research organisms

Policy information about studies involving animals; ARRIVE guidelines recommended for reporting animal research, and Sex and Gender in Research

Laboratory animals

Animals were housed in vivarium under 12:12 light/dark cycle conditions with ad libitum access to food and water. Temperature ranged from 21 to 23 °C and humidity ranged from 30% to 70%. Male and female mice were used with a mixed B6/129 background for all experiments at embryonic (E13.5-E15) and adult (P40-P150) stages. WT, Opn4Cre/+ (RRID:IMSR_JAX:035925). Brn3bcKOAP (RRID: IMSR_JAX:010559). Opn4CreERT2/+ (RRID:IMSR_JAX:035926). Rpl22HA (RRID: IMSR_JAX:029977). R26R-EYFP (RRID:IMSR_JAX:006148).

Wild animals

n/a

Reporting on sex

Male and Females were included in all experiments. Sex was not considered as a biological variable in the study design or analysis. Data were not disaggregated by sex, as the experiments were not powered to detect sex differences.

Field-collected samples

n/a

Ethics oversight

All procedures were approved by the Animal Care and Use Committee at Northwestern University. Protocol number: IS00003845

Note that full information on the approval of the study protocol must also be provided in the manuscript.

Plants

Seed stocks	n/a
Novel plant genotypes	n/a
Authentication	n/a
